# A novel frameshift mutation in the *EDA* gene in an Iranian patient affected by X-linked hypohidrotic ectodermal dysplasia

**DOI:** 10.1186/s11658-019-0174-9

**Published:** 2019-08-19

**Authors:** Marzieh Rahbaran, Maryam Hassani Doabsari, Simindokht Salavitabar, Neda Mokhberian, Ziba Morovvati, Saeid Morovvati

**Affiliations:** 1 Islamic Azad Tehran Medical Sciences University, Tehran, Iran; 2 Department of biotechnology, School of Advanced Technology in Medicine, Shahid Beheshti University of Medical Sciences, Tehran, Iran; 3 Department of Medical Genetics, Faculty of Advanced Medical Technologies, Golestan University of Medical Sciences, Gorgan, Iran; 4 Human Genetics Research Center, Baqiyatallah University of Medical Sciences, Mollasadra St, Tehran, Iran

**Keywords:** *EDA*, Gene, Hypohidrotic, Ectodermal, Dysplasia

## Abstract

**Purpose:**

Ectodermal dysplasias are characterized by developmental abnormalities in ectodermal structures. Hypohidrotic ectodermal dysplasias (HED) are the most common subtype. They are most commonly inherited via X-linked recessive routes. We report on a novel ectodysplasin-A (*EDA*) mutation that is expected to be involved in pathogenesis of HED.

**Methods:**

Hypohidrotic ectodermal dysplasia genes, including *EDA*, *EDAR* and *EDARADD*, were analyzed using next-generation sequencing (NGS). The detected mutation on the *EDA* gene was confirmed in the patient and his mother using Sanger sequencing.

**Results:**

The patient presented with adontia, absence of gum development, hyperthermia and hypohidrosis. Our genetic analysis of the patient revealed a novel frameshift hemizygous mutation (c.898_924 + 8del35ins4CTTA) on the *EDA* gene. The patient’s mother showed a mild HED phenotype. Direct sequencing of the *EDA* gene in the region where her son had the mutation showed the same mutation in a heterozygous state.

**Conclusion:**

We identified a novel frameshift mutation in the *EDA* gene in an Iranian patient affected by X-linked HED. The difference between our patient’s symptoms and those recorded for some previous subjects may be due to the differences in the mutations involved.

**Electronic supplementary material:**

The online version of this article (10.1186/s11658-019-0174-9) contains supplementary material, which is available to authorized users.

## Introduction

Ectodermal dysplasias (EDs) are a group of disorders characterized by developmental abnormalities in at least 2 of the following 4 ectodermal structures: nails, teeth, hair and sweat glands [1]. There are 2 major types of this disorder. The hypohidrotic–anhidrotic type, which is also termed as Christ-Siemens-Touraine syndrome, is characterized by hypotrichosis (skin, hair and nail anomalies), either hypodontia or anodontia, and hypohidrosis (partial or total absence of eccrine sweat glands). Other features include frontal bossing, a saddle-shaped nose, and everted lips. The hidrotic type is distinguished by hypotrichosis, ungual dystrophy, and hyperkeratosis of the palms and soles [2, 3].

Hypohidrotic ectodermal dysplasia (HED) is the most common subtype. Its incidence is estimated to be 1 per 100,000 births [4, 5]. Common symptoms in subjects with HED are a reduced number of teeth and sweat glands, reduced secretion of saliva, sparse and thin hair, and dry skin. Other clinical manifestations are dryness of airways and mucous membranes, presumably due to the defective development of exocrine glands. HED can also be associated with dysmorphic facial features, such as a prominent forehead, dark, hyper keratinized skin around the eyes, an everted nose and prominent lips [6].

HED is most commonly an X-linked recessive disorder and is rarely seen to be inherited via the autosomal recessive or dominant routes [7]. Therefore, it is observed in more males than females [8].

HED may result from defects in any of three interacting proteins: ectodysplasin-A (EDA), EDA receptor (EDAR) or EDAR-associated death domain (EDARADD) [9]. The molecular basis of X-linked hypohidrotic ectodermal dysplasia (XLHED) involves disruption in EDA protein [1].

The EDA protein is a type II transmembrane protein in the TNF superfamily [10]. It includes a transmembrane domain, an N-terminal intracellular domain, an extracellular domain, and a C-terminal domain containing the TNF homology domain. To be functionally active, EDA protein should be cleaved and released from cells where it forms a trimer that binds to the EDA receptor (EDAR) protein and activates it. EDA is cleaved at a special site referred to as the furin cleavage site. Any mutation at this site lead to an inability to form the active EDA trimer, resulting in disease. The *EDA* gene is comprised of 8 exons and several isoforms exist due to alternative splicing [1].

Here, we report on a novel frameshift *EDA* gene mutation that leads to the early termination of amino acid production. This is expected to affect the function of the EDA protein.

## Material and methods

**Fig. 1:**
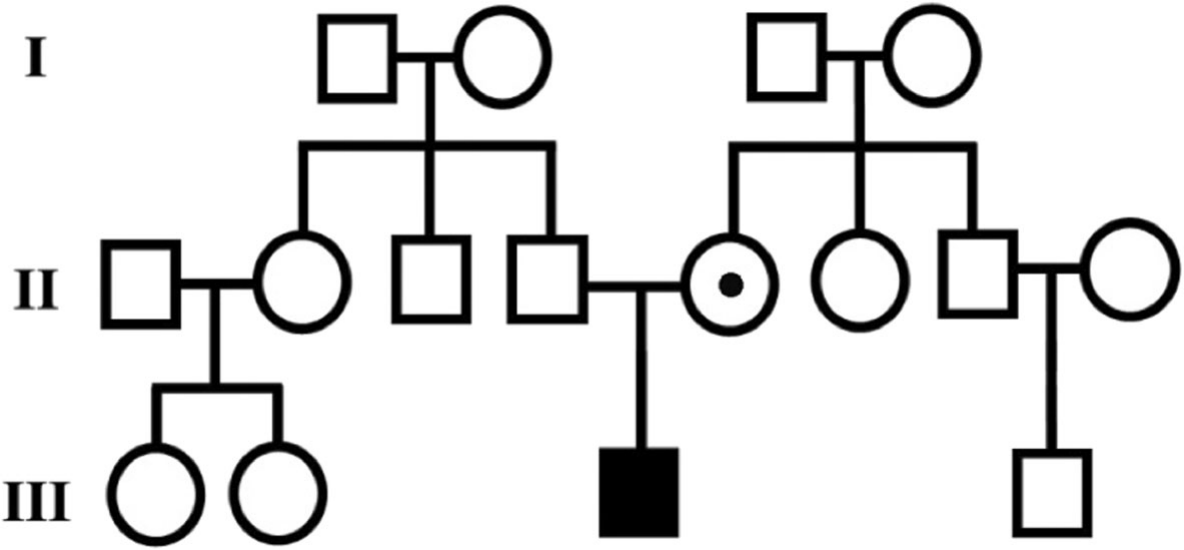
Family pedigree of the patient. The black square represents the patient. Patient's mother is a carrier of familial mutation

The study subject is an 8-year old boy. His disease was diagnosed when he was 7 years old. In this study, after genetic counseling and charting of the familial pedigree (Fig. 1), the patient was examined for the genetic causes of his disease.

After obtaining informed consent, 5 ml samples of peripheral blood were collected from the patient and his mother in EDTA-containing tubes. Genomic DNA was isolated from these samples using a standard phenol–chloroform DNA extraction method [11]. Genomic DNA sequencing for the patient was performed using a Nimblegen chip capturing the hypohidrotic ectodermal dysplasia genes, including *EDA*, *EDAR*, and *EDARADD*, followed by next-generation sequencing (Additional file 1). The test platform examined > 95% of the target gene with a sensitivity > 99%. Detected variations include single point mutations and small indels (within 20 bp). The analytical sensitivity and specificity of the NGS method used here to detect single point mutations and small indels (within 20 bp) are assumed to be > 95%.

The discovered variant is absent from dbSNP, Hapmap, 1000-genome, BGI’s and our local databases. Multiple lines of in silico computational analysis, namely Mutation Taster (disease causing), PhyloP (score: 5.176), PhCons (score: 1), CADD Raw (score: 4.46) and CADD PHRED (score: 33), support the deleterious effect of this variant on the gene product.

The detected mutation on the *EDA* gene was confirmed in the patient and his mother using Sanger sequencing with the forward primer: 5′-TTC TCT GCT TTC AAA TGC TCT TC-3′ and the reverse primer: 5′-CAG GAA GTT AGC CAT TGG ATG-3′. PCR was carried out in a total volume of 25 μl containing 200 ng DNA template, 20 pM of each of the primers, 3 mM MgCl2, and 400 μM of each of dNTP and Taq DNA polymerase 2.0 U. DNA amplification was performed in a Mastercycler 5330 (Eppendorf). The amplification conditions were 94 °C for 2 min, followed by 35 cycles of 94 °C for 30 s, 55 °C for 30 s and 72 °C for 30 s, with a final extension at 72 °C for 7 min. The amplified PCR products were analyzed using Sanger sequencing.

## Results

Clinical examination of the patient, an 8-year old boy, revealed the typical features of HED. The pedigree of the XLHED family was charted based on the clinical symptoms (Fig. 1). The represented family is one child and his father and mother. The patient presented with adontia, the absence of gum development, hyperthermia and hypohidrosis. His skin was dry and wrinkled with no nail dystrophy and responded well to topical moisturizers. The scalp hair and eyelashes were sparse, thin, and lightly pigmented, and the patient had no eyebrows. He suffered from recurrent infections in childhood but is now less sensitive to infection. Due to hypertrophic tonsils, he has difficulty breathing. The patient shows delays in physical development, such as walking, sitting and speaking, and considerable intellectual disability.

**Fig. 2:**
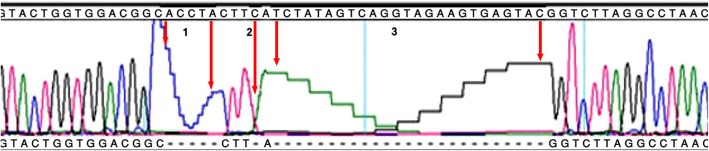
gDNA direct sequencing of the region where the NGS test detected the c.898_924 + 8del35ins4CTTA mutation on EDA gene (NM_001399) in the affected child

**Fig. 3:**
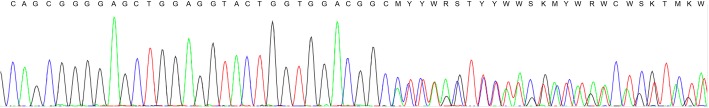
gDNA direct sequencing for the mother of affected child using Sanger sequencing method. Chromatogram is showing the frameshift mutation

Genetic analysis of the patient revealed a novel frameshift hemizygous mutation (c.898_924 + 8del35ins4CTTA) on the *EDA* gene (NM_001399.5) (Fig. 2). The patient’s mother showed a mild HED phenotype. She presented with peg-shaped oligodontia with complete gum. Direct sequencing of the *EDA* gene in the region where her son had the mutation showed the same mutation in a heterozygous state (Fig. 3). Therefore, the patient has inherited HED from his carrier mother. The father and his family showed no signs or symptoms of the disease. The list of the variants found in the *EDA*, *EDAR* and *EDARADD* genes with their detailed descriptions are explained in Table 1. Previously reported pathogenic and likely pathogenic mutations in the *EDA*, *EDAR* and *EDARADD* genes are listed in Additional file 2.

**Table 1:** List of the variants identified on EDA, EDAR and EDARADD genes in the affected child investigated by NGS method

Gene name	Variant name	RS-ID	Frequency in			
			dbSNP	Hapmap	1000-genome	BGI’s
*EDA*	c.898_924+8del35ins4 (Hemizygous)	Novel	-	-	0	0
*EDARADD*	p.Met9Ile (Hom)	rs966365	0.59	0.881	0.62	0.8889
*EDAR*	p.Cys352Cys (Het)	rs12623957	0.352	0.066	0.2619	0.0525
*EDAR*	p.Ser250Ser (Het)	rs260632	0.13	0.007	0.1218	0.089

## Discussion

Hypohidrotic ectodermal dysplasia (HED) is an X-linked condition that is considered to be the most common type of ectodermal dysplasia (ED). It can be inherited as autosomal recessive or autosomal dominant pattern. In X-linked HED, the affected patients are most often hemizygous male subjects since males have only one X chromosome and one altered copy of the gene in each cell is sufficient to cause the disorder [8].

In X-linked recessive disorders, the disease in females usually only results from a mutation in both copies of the gene. However, in X-linked HED, some heterozygous females show a mild phenotype of the disease. They have few missing or abnormal teeth, sparse hair and some problems with sweat gland function [4]. These female patients are referred to as manifesting heterozygous individuals. This phenomenon is caused by random X-inactivation [7]. This generally occurs early in development, after approximately 15 to 16 days’ gestation, when the embryo consists of approximately 5000 cells. The inactive X chromosome exists in a condensed form during interphase, when it appears as a darkly staining mass of ‘sex chromosome’, or a Barr body.

The mild presentation of the disease in the patient’s mother in this study can thus be explained by random X-inactivation leaving the mutant X chromosome as an active chromosome in a portion of her cells.

The gene responsible for X-linked HED, *EDA*, is located at Xq12-q13.1. It encodes EDA, which is important for the development of several organs and structures derived from the ectoderm, such as the skin, hair and nails [12]. Evidence shows that ectodysplasin-A is essential in numerous pathways that involve ectodermal–mesodermal interactions during embryogenesis. Defects in the molecular structure of ectodysplasin-A may disrupt the action of enzymes that are required for the normal development of the ectoderm [13]. Earlier research has identified a number of mutations that result in XLHED, including small and large deletions [14, 15], insertions [16, 17], frameshifts [16], and substitutions [18–22]. Although the type of mutation shows no obvious correlation with the phenotype and severity of disease, especially for heterozygous carriers [23], some studies have suggested that the variation in the phenotype of XLHED is associated with different mutations in the *EDA* gene. The genetic variability in this condition may lead to variability in its characteristics, including different dental phenotypes [5].

Khabour et al. identified a missense mutation (c.463C > T) in the *EDA* gene in a Jordanian family. This mutation brings about an arginine-to-cysteine change in the extracellular domain of ectodysplasin-A. The phenotype of an affected 11-year old boy with this mutation included heat intolerance, sparse hair, oligodontia, speech problems, and damaged eccrine glands resulting in reduced sweating [4].

In 2013, Yin et al. reported a frameshift mutation, c.573–574insT, in the *EDA* gene. The insertion induced a frameshift from amino acid 192 and caused transcription to stop at amino acid 239. Their patients had sparse hair, eyelashes and eyebrows; misshapen or missing teeth; decreased sweating and salivary secretions; and characteristic facial features including prominent forehead, narrow and short maxillary regions, small cranial length, and depressed nasal root and bridge [23].

In 2017, Savasta et al. investigated a male and his family with a novel pathogenic missense mutation, c.158 T > A, in a hemizygosity state in exon 1 of the *EDA* gene. The case had a delayed dental eruption; sparse, fine and stiff blond scalp hair; reduced eyebrows; and periorbital hyperpigmentation. The features of his face included frontal bossing and chin prominence with a saddle nose, maxillary hypoplasia, and protuberant lips. His midface was depressed, and the lower third of the face appeared smaller due to lack of alveolar bone development. As with our study subject, sparse, thin and lightly pigmented scalp hair and eyelashes with no eyebrows were reported. Also similarly to our case, the skin was dry and wrinkled with no nail dystrophy and it responded well to topical moisturizers [24].

In 2015, Xue et al. revealed a report of a novel missense mutation (c.878 T > G) in the *EDA* gene in a 21-year old man. The case had sparse hair and eyebrows, thin and dry skin, and characteristic facial features, like frontal bossing, a saddle nose, prominent lips, a juga chin and maxillary hypoplasia. These features are similar to our patient’s. As mentioned, our patient also had dry and wrinkled skin, sparse scalp hair, sparse, thin and lightly pigmented eyelashes and no eyebrows [25].

In 2012, Liu et al. reported a novel mutation in exon 8 of the *EDA* gene (c.1061 T > C (p.Leu354Pro)) in a patient affected with XLHED in a Chinese family. Their patient shared absent eyebrows, sparse and thin hair, and misshapen or missing teeth with ours [26].

Some features in our patient, including delays in development and intellectual disability, have not been previously reported in XLHED patients and may be caused by further mutations not located within the *EDA* gene.

In conclusion, we identified a novel frameshift mutation in the *EDA* gene in an Iranian patient affected by XLHED. Although there is no existing paper reporting on this mutation, the frameshift mutation in the end of exon 8 (c.898_924 + 8del35ins4CTTA) on the *EDA* gene makes an early termination in amino acid production (truncated protein), which would be expected to affect the protein’s function.

**Fig. 4:**
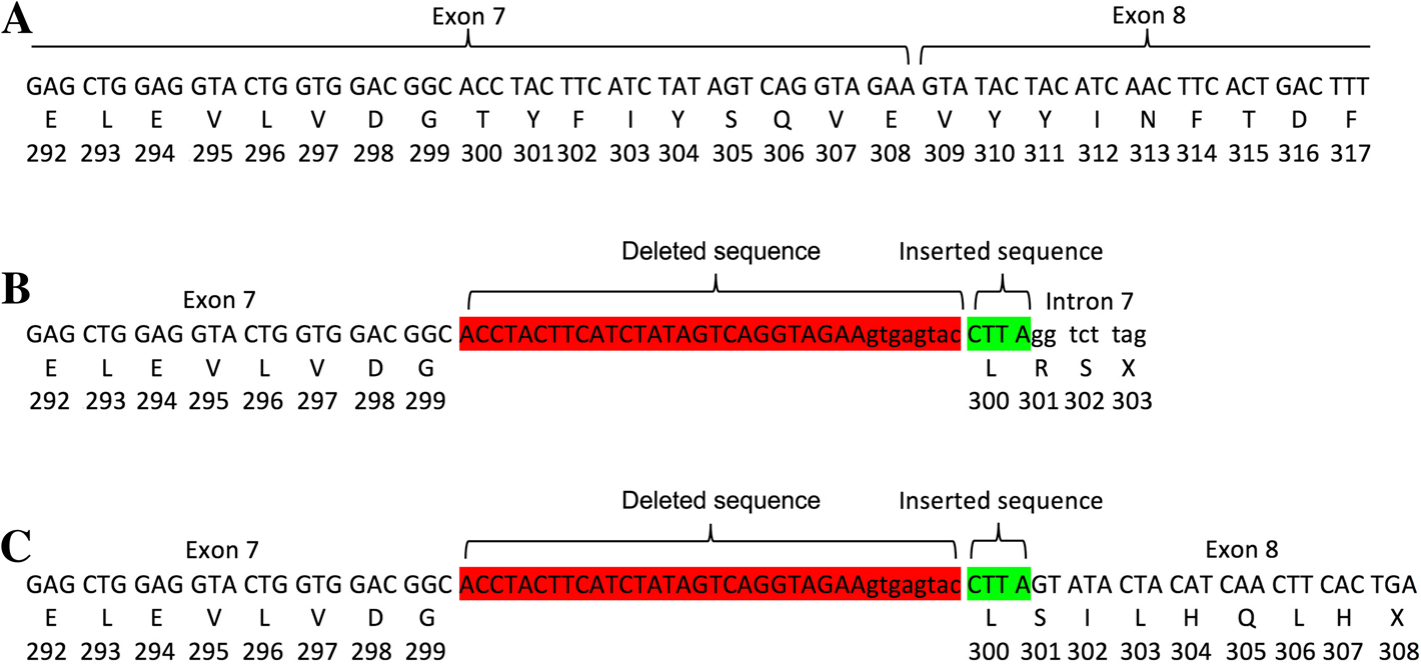
The frameshift mutation in the end of exon 8 (c.898_924 + 8del35ins4CTTA) on EDA gene makes an early termination in amino acid production, which is expected

Although this mutation has not been verified at the cDNA level, it may result in an early termination of amino acid production at codon 302, if the translation is continued in intron 7, (Fig. 4b) or codon 307, if the translation is continued in exon 8 (Fig. 4c). This would result in the complete lack of exon 8 and the C-terminal tumor necrosis factor homology domain in the extracellular domain of the ectodysplasin-A protein (Fig. 4).

## Additional files

Additional file 1: The report of NGS panel test of the patient. (PDF 505 kb)

Additional file 2: List of previously reported mutations in EDA, EDAR and EDARADD genes. (PDF 1275 kb)

## Data Availability

The datasets used and/or analyzed during this study are available from the author for correspondence upon reasonable request. The patient’s parents agreed to the publication of data related to their issue. Information that supports the results of this study can be found in supplementary attachment files.

## Abbreviations

EDA: Ectodysplasin-A
EDAR: EDA receptor
EDARADD: EDAR-associated death domain
EDs: Ectodermal dysplasias
HED: Hypohidrotic ectodermal dysplasias
NGS: Next-generation sequencing
XLHED: X-linked hypohidrotic ectodermal dysplasia
