# Supplementary material for: A novel frameshift mutation in the EDA gene in an Iranian patient affected by X-linked hypohidrotic ectodermal dysplasia
Source: Cell Mol Biol Lett. 2019 Aug 19;24:54. doi: 10.1186/s11658-019-0174-9 (PMC6700831; doi:10.1186/s11658-019-0174-9)
Supplement: Supplementary file 1 — The report of NGS panel test of the patient. (PDF 505 kb) [file 11658_2019_174_MOESM1_ESM.pdf]

**Interpretation:** A hemizygous mutationc.898_924+8del35ins4 on gene ***EDA*** of sample 13D0280106 has been detected. Although there is no paper reporting this mutation, the frameshift mutation makes the early termination of amino acid production, which is expected to affect the protein's function. As the ***Hypohidrotic Ectodermal Dysplasia*** is inherited in X-linked manner, the hemizygous mutation, c.898_924+8del35ins4 on gene ***EDA,*** is possible pathogenic mutation of sample 13D0280106.

**Recommendation:** The mother should be tested by Sanger sequencing for the mutation, c.898_924+8del35ins4, which are suggested.

**Mutation(s) on *EDA* genes related to clinical phenotypes**

| Mutation Name | Nucleic Acid Alternation | Amino Acid Alternation | Mutation location | Homozygous/ Heterozygous/ Hemizygous | Reference |
|---|---|---|---|---|---|
| c.898_924+8del35ins4 | c.898_924+8del35ins4 | - | CDS7/EX7 | Hemizygous | - |

**List of the variant(s) identified on *EDA* gene**

| Variant Name | RS-ID | Frequency in | | | |
|---|---|---|---|---|---|
| | | dbSNP | Hapmap | 1000-genome | BGI's |
| c.898_924+8del35ins4 (Hemizygous) | novel | - | - | 0 | 0 |

**List of the variant(s) identified on *EDARADD* gene**

| Variant Name | RS-ID | Frequency in | | | |
|---|---|---|---|---|---|
| | | dbSNP | Hapmap | 1000-genome | BGI's |
| p.Met9Ile(Hom) | rs966365 | 0.59 | 0.881 | 0.62 | 0.8889 |

**List of the variant(s) identified on *EDAR* gene**

| Variant Name | RS-ID | Frequency in | | | |
|---|---|---|---|---|---|
| | | dbSNP | Hapmap | 1000-genome | BGI's |
| p.Cys352Cys(Het) | rs12623957 | 0.352 | 0.066 | 0.2619 | 0.0525 |
| p.Ser250Ser(Het) | rs260632 | 0.13 | 0.007 | 0.1218 | 0.089 |


BGI Clinical Laboratories (Shenzhen) Co., Ltd
Beishan Industrial Zone, Yantian District, Shenzhen P.R. China 518083
email: birth-health@genomics.cn website: www.genomics.cn
Fax: +86 755 2527 3780

dbSNP: SNP frequency in the dbSNP database

Hapmap: SNP frequency in Asia population in the Hapmap database

1000-genome: SNP frequency in all the samples from the 1000 Genome Project

BGI's: SNP frequency in BGI's local >200people genome database

## Reference

[1]J Timothy Wright, DDS, MS, Dorothy K Grange, MD, and Mary K Richter.HypohidroticEctodermal Dysplasia.GeneReviews (http://www.ncbi.nlm.nih.gov/books/NBK1112/)

## Test Information

| | |
|---|---|
| Disease | ***Hypohidrotic Ectodermal Dysplasia***, Autosomal Recessive/Dominant, X-linked |
| Genes | ***EDA***(NM_001399) :*ex1-8*;***EDAR***(NM_022336) :ex*1-12*;***EDARADD***(NM_145861) :ex*1-6* |

## Sequencing Quality Report

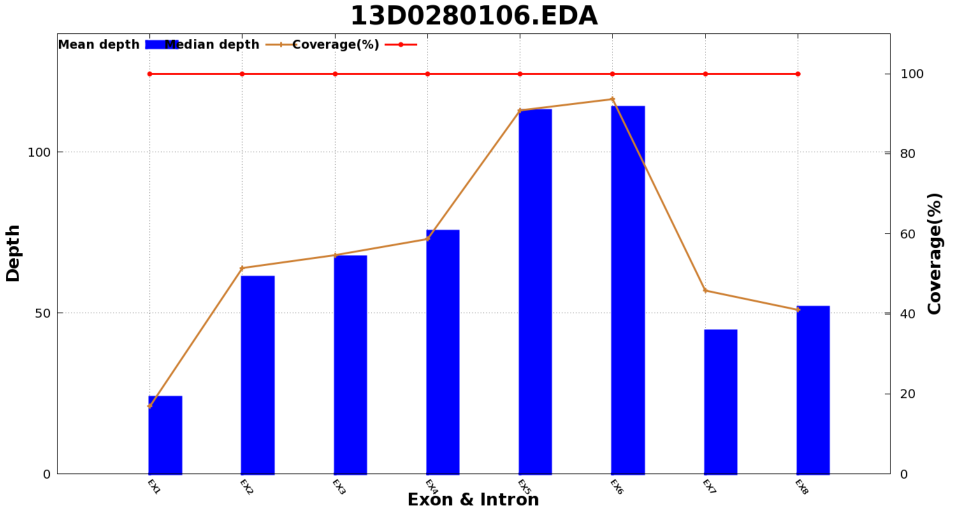


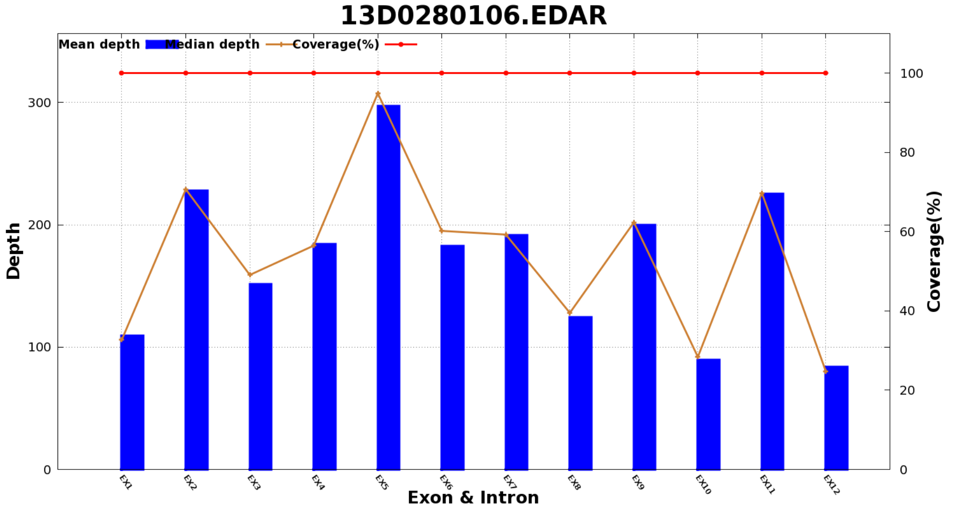




BGI Clinical Laboratories (Shenzhen) Co., Ltd
Beishan Industrial Zone, Yantian District, Shenzhen P.R. China 518083
email: birth-health@genomics.cn website: www.genomics.cn
Fax: +86 755 2527 3780

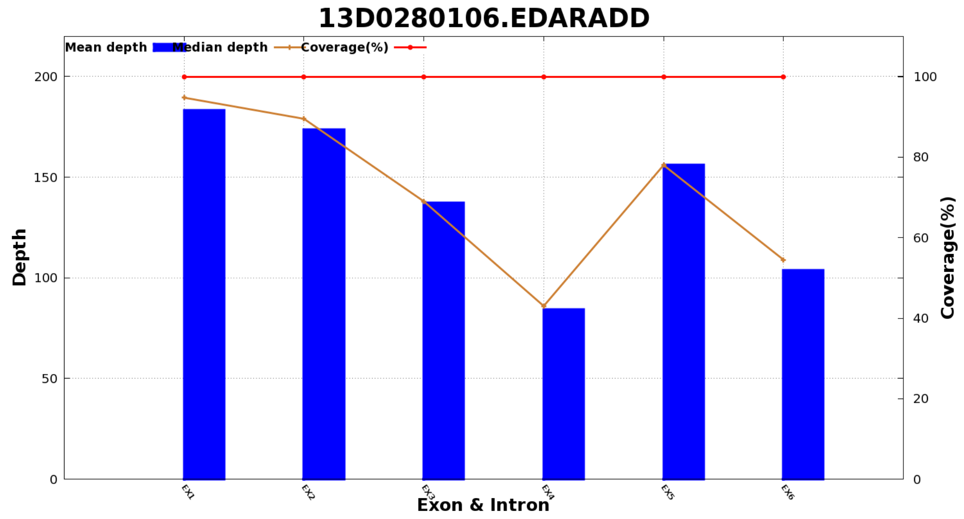


On average 99.9% of base pairs with>100×coverage were successfully detected.

The average of sequencing depth approximates to the sequencing depth median of all exons, which means good randomicity of sequencing.

**Methodology**

The Genetic Sequencing Test is performed using a custom designed Nimblegen chip capturing the genes of interest followed by Next Generation Sequencing. In general, the test platform examined >95% of the target gene with sensitivity > 99%. Point mutation, micro-insertion, deletion and duplication (<20bp) can be simultaneously detected. Bioinformatic analysis of the sequencing results using international mutation and polymorphism databases as well as our self-developed local database provides association of the mutations/variations with the clinical conditions. For novel mutations, prediction of the consequence of such mutation(s) will be provided. More detail information regarding the mutation(s) and clinical conditions are available at http://sdmd.genomics.org.cn.

Yun Li、Kun Wang
Genetic Test Operator, BGI

Date: 2014-02-01

Ming Qi
PhD, American Board of Medical Genetics certified Clinical Molecular Geneticist, Fellow of the American College of Medical Genetics, BGI Health

Date: 2014-02-17

This report is specific to the tested sample, and cannot be used for other purposes.



BGI Clinical Laboratories (Shenzhen) Co., Ltd
Beishan Industrial Zone, Yantian District, Shenzhen P.R. China 518083
email: birth-health@genomics.cn website: www.genomics.cn
Fax: +86 755 2527 3780

Data listed above is generated from the laboratory standard testing procedure, and is only used for clinical reference.

The test results are obtained using Next Generation Sequencing. Sanger Sequencing on the identified mutation(s) for validation is highly recommended.

BGI Clinical Laboratories reserve the rights of final explanation of this report. For inquiry, please kindly contact us within 7 days after receiving the test report.

--- End of Report ---



BGI Clinical Laboratories (Shenzhen) Co., Ltd
Beishan Industrial Zone, Yantian District, Shenzhen P.R. China 518083
email: birth-health@genomics.cn website: www.genomics.cn
Fax: +86 755 2527 3780
