# Supplementary material for: A novel frameshift mutation in the EDA gene in an Iranian patient affected by X-linked hypohidrotic ectodermal dysplasia
Source: Cell Mol Biol Lett. 2019 Aug 19;24:54. doi: 10.1186/s11658-019-0174-9 (PMC6700831; doi:10.1186/s11658-019-0174-9)
Supplement: Supplementary file 2 — List of previously reported mutations in EDA, EDAR and EDARADD genes. (PDF 1275 kb) [file 11658_2019_174_MOESM2_ESM.pdf]

EDAR Gene

Variant ID	Chr: bp	Alleles	Clinical Significance	Consequence Type	Resulting amino acid(s)	SIFT	PolyPhen	CADD	REVEL	Meta LR	Mutation Assessor
rs886041005	2:108896970	A/**C**/T		missense variant	C/W	0	0.999	23	0.612	0.543	0.144
rs886041005	2:108896970	A/C/**T**		stop gained	C/*	-	-	-	-	-	-
rs121908453	2:108896995	C/T		missense variant	R/Q	0.01	0.998	25	0.733	0.67	0.144
rs1064793107	2:108897025	T/C		missense variant	E/G	0.01	0.67	32	0.65	0.477	0.065
rs917638291	2:108897091	A/G		missense variant	I/T	0	0.998	28	0.812	0.733	0.144
rs747806672	2:108897110	C/T		missense variant	G/S	0	1	27	0.792	0.714	0.144
rs121908454	2:108897130	C/T		missense variant	R/H	0.01	0.999	33	0.806	0.674	0.144
rs886039348	2:108897133	C/**A**/T		missense variant	W/L	0.12	0.998	25	0.921	0.959	0.144
rs886039348	2:108897133	C/A/**T**		stop gained	W/*	-	-	-	-	-	-
rs886039564	2:108897181	C/T		missense variant	R/Q	0.03	0.992	28	0.74	0.732	0.144
rs121908452	2:108897182	G/A		stop gained	R/*	-	-	-	-	-	-

rs121908456	2:108897194	C/**A**/T		stop gained	E/*	-	-	-	-	-	-
rs121908456	2:108897194	C/A/**T**		missense variant	E/K	**0**	**0.986**	**25**	**0.663**	**0.837**	**0.631**
rs199544410	2:108907920	G/T		stop gained	C/*	-	-	-	-	-	-
rs1064793684	2:108907934-108907940	AGCCCTG/CGGGCTCCTCATCA		stop gained<br><br>frameshift variant	QGS/**GARX	-	-	-	-	-	-
rs797044437	2:108910459	C/T		splice donor variant	-	-	-	-	-	-	-
rs797044436	2:108910784-108910787	TCTT/-		frameshift variant	KE/X	-	-	-	-	-	-
rs368841777	2:108923367	C/T		splice donor variant	-	-	-	-	-	-	-
rs121908455	2:108929225	T/G		missense variant	D/A	**0**	**0.994**	**24**	**0.923**	**0.896**	**0.859**
rs557166582	2:108929262	G/**A**/T		missense variant	R/W	**0**	**0.936**	**33**	**0.86**	**0.896**	**0.803**
rs557166582	2:108929262	G/A/**T**		synonymous variant	R	-	-	-	-	-	-
rs1060499610	2:108929270	C/-		frameshift variant	G/X	-	-	-	-	-	-
rs121908450	2:108929288	C/T			R/H	**0.01**	**0.991**	**27**	**0.799**	**0.837**	**0.808**

				missense variant							
rs780424781	2:108929289	G/A		missense variant	R/C	0.01	0.993	32	0.795	0.82	0.808
rs121908451	2:108929295	A/G		missense variant	C/R	0	0.991	26	0.951	0.966	0.888
rs777022647	2:108929342	C/T		missense variant	C/Y	0	0.991	25	0.92	0.97	0.896
rs797044435	2:108930963	C/T		splice donor variant	-	-	-	-	-	-	-
rs886041005	2:108896970	A/**C**/T		missense variant	C/W	0	0.999	23	0.612	0.543	0.144
rs886041005	2:108896970	A/C/**T**		stop gained	C/*	-	-	-	-	-	-
rs121908453	2:108896995	C/T		missense variant	R/Q	0	0.998	25	0.733	0.67	0.144
rs1064793107	2:108897025	T/C		missense variant	E/G	0	0.735	32	0.65	0.477	0.065
rs917638291	2:108897091	A/G		missense variant	I/T	0	0.998	28	0.812	0.733	0.144
rs747806672	2:108897110	C/T		missense variant	G/S	0	1	27	0.792	0.714	0.144
rs121908454	2:108897130	C/T		missense variant	R/H	0	0.999	33	0.806	0.674	0.144

rs886039348	2:108897133	C/**A**/T		missense variant	W/L	0	0.998	25	0.921	0.959	0.144
rs886039348	2:108897133	C/A/**T**		stop gained	W/*	-	-	-	-	-	-
rs886039564	2:108897181	C/T		missense variant	R/Q	0.01	0.992	28	0.74	0.732	0.144
rs121908452	2:108897182	G/A		stop gained	R/*	-	-	-	-	-	-
rs121908456	2:108897194	C/**A**/T		stop gained	E/*	-	-	-	-	-	-
rs121908456	2:108897194	C/A/**T**		missense variant	E/K	0	0.986	25	0.663	0.837	0.631
rs199544410	2:108907920	G/T		stop gained	C/*	-	-	-	-	-	-
rs1064793684	2:108907934-108907940	AGCCCTG/CGGGCTCCTCATCA		stop gained<br>frameshift variant	QGS/**GARX	-	-	-	-	-	-
rs797044437	2:108910459	C/T		splice donor variant	-	-	-	-	-	-	-
rs797044436	2:108910784-108910787	TCTT/-		frameshift variant	KE/X	-	-	-	-	-	-
rs368841777	2:108923367	C/T		splice donor variant	-	-	-	-	-	-	-
rs121908455	2:108929225	T/G		missense variant	D/A	0	0.996	24	0.923	0.896	0.859

rs557166582	2:108929262	G/**A**/T		missense variant	R/W	0	0.965	33	0.86	0.896	0.803
rs557166582	2:108929262	G/A/**T**		synonymous variant	R	-	-	-	-	-	-
rs1060499610	2:108929270	C/-		frameshift variant	G/X	-	-	-	-	-	-
rs121908450	2:108929288	C/T		missense variant	R/H	0.01	0.996	27	0.799	0.837	0.808
rs780424781	2:108929289	G/A		missense variant	R/C	0.01	0.996	32	0.795	0.82	0.808
rs121908451	2:108929295	A/G		missense variant	C/R	0	0.996	26	0.951	0.966	0.888
rs777022647	2:108929342	C/T		missense variant	C/Y	0	0.996	25	0.92	0.97	0.896
rs797044435	2:108930963	C/T		splice donor variant	-	-	-	-	-	-	-
rs886041005	2:108896970	A/**C**/T		missense variant	C/W	0	0.999	23	0.612	0.543	0.144
rs886041005	2:108896970	A/C/**T**		stop gained	C/*	-	-	-	-	-	-
rs121908453	2:108896995	C/T		missense variant	R/Q	0	0.998	25	0.733	0.67	0.144
rs1064793107	2:108897025	T/C		missense variant	E/G	0	0.735	32	0.65	0.477	0.065

rs917638291	2:108897091	A/G		missense variant	I/T	0	0.998	28	0.812	0.733	0.144
rs747806672	2:108897110	C/T		missense variant	G/S	0	1	27	0.792	0.714	0.144
rs121908454	2:108897130	C/T		missense variant	R/H	0	0.999	33	0.806	0.674	0.144
rs886039348	2:108897133	C/**A**/T		missense variant	W/L	0	0.998	25	0.921	0.959	0.144
rs886039348	2:108897133	C/A/**T**		stop gained	W/*	-	-	-	-	-	-
rs886039564	2:108897181	C/T		missense variant	R/Q	0.01	0.992	28	0.74	0.732	0.144
rs121908452	2:108897182	G/A		stop gained	R/*	-	-	-	-	-	-
rs121908456	2:108897194	C/**A**/T		stop gained	E/*	-	-	-	-	-	-
rs121908456	2:108897194	C/A/**T**		missense variant	E/K	0	0.986	25	0.663	0.837	0.631
rs199544410	2:108907920	G/T		stop gained	C/*	-	-	-	-	-	-
rs1064793684	2:108907934-108907940	AGCCCTG/CGGGCTCCTCATCA		stop gained<br>frameshift variant	QGS/**GARX	-	-	-	-	-	-
rs797044437	2:108910459	C/T		splice donor variant	-	-	-	-	-	-	-

rs797044436	2:108910784-108910787	TCTT/-		frameshift variant	KE/X	-	-	-	-	-	-
rs368841777	2:108923367	C/T		splice donor variant	-	-	-	-	-	-	-
rs121908455	2:108929225	T/G		missense variant	D/A	**0**	**0.996**	**24**	**0.923**	**0.896**	**0.859**
rs557166582	2:108929262	G/**A**/T		missense variant	R/W	**0**	**0.965**	**33**	**0.86**	**0.896**	**0.803**
rs557166582	2:108929262	G/A/**T**		synonymous variant	R	-	-	-	-	-	-
rs1060499610	2:108929270	C/-		frameshift variant	G/X	-	-	-	-	-	-
rs121908450	2:108929288	C/T		missense variant	R/H	**0.01**	**0.996**	**27**	**0.799**	**0.837**	**0.808**
rs780424781	2:108929289	G/A		missense variant	R/C	**0.01**	**0.996**	**32**	**0.795**	**0.82**	**0.808**
rs121908451	2:108929295	A/G		missense variant	C/R	**0**	**0.996**	**26**	**0.951**	**0.966**	**0.888**
rs777022647	2:108929342	C/T		missense variant	C/Y	**0**	**0.996**	**25**	**0.92**	**0.97**	**0.896**
rs797044435	2:108930963	C/T		splice donor variant	-	-	-	-	-	-	-

EDARADD Gene

Variant ID	Chr: bp	Alleles	Clinical Significance	Consequence Type	Resulting amino acid(s)	SIFT	PolyPhen	CADD	REVEL	Meta LR	Mutation Assessor
rs954823206	1:236482418	G/A		stop gained	W/*	-	-	-	-	-	-
rs954823206	1:236482418	G/A		stop gained	W/*	-	-	-	-	-	-
rs954823206	1:236482418	G/A		stop gained	W/*	-	-	-	-	-	-
rs954823206	1:236482418	G/A		intron variantNMD transcript variant	-	-	-	-	-	-	-
rs879255553	1:236409275	G/A		splice donor variant	-	-	-	-	-	-	-
rs766500689	1:236427427	C/T		stop gained	R/*	-	-	-	-	-	-
rs121908116	1:236482366	T/G		missense variant	L/R	0	1	24	0.758	0.672	0.281
rs879255629	1:236482368	G/A		missense variant	D/N	0.34	0.986	26	0.369	0.524	0.202
rs397515575	1:236482403-236482408	AACGGT/-		inframe deletion	PTV/P	-	-	-	-	-	-
rs74315309	1:236482455	G/A		missense variant	E/K	0	0.999	27	0.491	0.294	0.281
rs879255553	1:236409275	G/A		splice donor variant	-	-	-	-	-	-	-
rs766500689	1:236427427	C/T		stop gained	R/*	-	-	-	-	-	-
rs121908116	1:236482366	T/G		missense variant	L/R	0	1	24	0.758	0.672	0.281

rs879255629	1:236482368	G/A		missense variant	D/N	**0.43**	**0.977**	**26**	**0.369**	**0.524**	**0.202**
rs397515575	1:236482403-236482408	AACGGT/-		inframe deletion	PTV/P	-	-	-	-	-	-
rs74315309	1:236482455	G/A		missense variant	E/K	**0**	**0.999**	**27**	**0.491**	**0.294**	**0.281**
rs879255553	1:236409275	G/A		splice donor variant	-	-	-	-	-	-	-
rs766500689	1:236427427	C/T		stop gained	R/*	-	-	-	-	-	-
rs879255553	1:236409275	G/A		splice donor variant	-	-	-	-	-	-	-
rs766500689	1:236427427	C/T		stop gained	R/*	-	-	-	-	-	-
rs121908116	1:236482366	T/G		missense variant	L/R	**0**	**1**	**24**	**0.758**	**0.672**	**0.281**
rs879255629	1:236482368	G/A		missense variant	D/N	**0.45**	**0.986**	**26**	**0.369**	**0.524**	**0.202**
rs397515575	1:236482403-236482408	AACGGT/-		inframe deletion	PTV/P	-	-	-	-	-	-
rs74315309	1:236482455	G/A		missense variant	E/K	**0**	**0.999**	**27**	**0.491**	**0.294**	**0.281**
rs879255553	1:236409275	G/A		splice donor variant<br>NMD transcript variant	-	-	-	-	-	-	-
rs766500689	1:236427427	C/T		stop gained<br>NMD transcript variant	R/*	-	-	-	-	-	-
rs121908116	1:236482366	T/G		intron variantNMD transcript variant	-	-	-	-	-	-	-
rs879255629	1:236482368	G/A		intron variantNMD transcript variant	-	-	-	-	-	-	-

rs397515575	1:236482403-236482408	AACGGT/-		intron variantNMD transcript variant	-	-	-	-	-	-	-
rs74315309	1:236482455	G/A		intron variantNMD transcript variant	-	-	-	-	-	-	-

EDA Gene

Variant ID	Chr: bp	Alleles	Clinical Significance	Consequence Type	Resulting amino acid(s)	SIFT	PolyPhen	CADD	REVEL	Meta LR	Mutation Assessor
rs397516659	X:69616310	T/C		start lost	M/T	0	0.733	22	0.689	0.919	-
rs132630310	X:69616375	C/T		stop gained	Q/*	-	-	-	-	-	-
rs727505089	X: between 69616443 & 69616444	-/GGGT		frameshift variant	-/GX	-	-	-	-	-	-
rs397516657	X:69616472	T/A		missense variant	L/Q	0	0.999	27	0.655	0.906	0.144
rs1131692034	X:69616488	C/A		stop gained	C/*	-	-	-	-	-	-
rs132630308	X:69616489	T/C		missense variant	Y/H	0	0.998	27	0.866	0.957	0.246
rs132630318	X:69616491	C/G		stop gained	Y/*	-	-	-	-	-	-
rs132630311	X:69616495	G/A		missense variant	E/K	0.01	0.971	28	0.833	0.923	0.18
rs132630319	X:69616501	C/G			R/G	0	0.999	25	0.872	0.963	0.246

				missense variant							
rs1057517971	X:69616553	G/-		frameshift variant	G/X	-	-	-	-	-	-
rs397516656	X: between 69616580 & 69616581	-/G		frameshift variant	S/RX	-	-	-	-	-	-
rs876657684	X: between 69616630 & 69616631	-/GGGC		frameshift variant	Q/RAX	-	-	-	-	-	-
rs397516660	X:69616637	C/A		stop gained	S/*	-	-	-	-	-	-
rs397516661	X:69616655	T/A		stop gained	L/*	-	-	-	-	-	-
rs727504537	X:69616705	G/A		missense variant	V/M	**0.41**	**0.804**	**27**	-	-	-
rs727504814	X:69616706	T/**C**/G		missense variant	V/A	**0.59**	**0.164**	**27**	-	-	-
rs727504814	X:69616706	T/C/**G**		missense variant	V/G	**0**	**0.009**	**27**	-	-	-
rs397516659	X:69616310	T/C		non coding transcript exon variant	-	-	-	-	-	-	-
rs132630310	X:69616375	C/T		non coding transcript exon variant	-	-	-	-	-	-	-
rs727505089	X: between 69616443 & 69616444	-/GGGT		non coding transcript exon variant	-	-	-	-	-	-	-

rs397516657	X:69616472	T/A		non coding transcript exon variant	-	-	-	-	-	-	-
rs1131692034	X:69616488	C/A		non coding transcript exon variant	-	-	-	-	-	-	-
rs132630308	X:69616489	T/C		non coding transcript exon variant	-	-	-	-	-	-	-
rs132630318	X:69616491	C/G		non coding transcript exon variant	-	-	-	-	-	-	-
rs132630311	X:69616495	G/A		non coding transcript exon variant	-	-	-	-	-	-	-
rs132630319	X:69616501	C/G		non coding transcript exon variant	-	-	-	-	-	-	-
rs1057517971	X:69616553	G/-		non coding transcript exon variant	-	-	-	-	-	-	-
rs397516656	X: between 69616580 & 69616581	-/G		non coding transcript exon variant	-	-	-	-	-	-	-
rs876657684	X: between 69616630 & 69616631	-/GGGC		non coding transcript exon variant	-	-	-	-	-	-	-
rs397516660	X:69616637	C/A		non coding transcript exon variant	-	-	-	-	-	-	-
rs397516661	X:69616655	T/A		non coding transcript exon variant	-	-	-	-	-	-	-
rs727504537	X:69616705	G/A			-	-	-	-	-	-	-

				splice donor variant non coding transcript variant							
rs727504814	X:69616706	T/**C**/G		splice donor variant non coding transcript variant	-	-	-	-	-	-	-
rs727504814	X:69616706	T/C/**G**		splice donor variant non coding transcript variant	-	-	-	-	-	-	-
rs397516662	X:69957087	C/T		non coding transcript exon variant	-	-	-	-	-	-	-
rs132630312	X:69957093	C/T		non coding transcript exon variant	-	-	-	-	-	-	-
rs132630313	X:69957096	C/T		non coding transcript exon variant	-	-	-	-	-	-	-
rs132630314	X:69957097	G/**A**/T		non coding transcript exon variant	-	-	-	-	-	-	-
rs132630314	X:69957097	G/A/**T**		non coding transcript exon variant	-	-	-	-	-	-	-
rs727504649	X:69957104	A/C		non coding transcript exon variant	-	-	-	-	-	-	-

rs876657640	X:69957107	A/T		non coding transcript exon variant	-	-	-	-	-	-	-
rs1057521131	X:69957124	G/C		non coding transcript exon variant	-	-	-	-	-	-	-
rs727505013	X:69957133	G/A		non coding transcript exon variant	-	-	-	-	-	-	-
rs397516659	X:69616310	T/C		start lost	M/T	0	0.057	22	0.689	0.919	-
rs132630310	X:69616375	C/T		stop gained	Q/*	-	-	-	-	-	-
rs727505089	X: between 69616443 & 69616444	-/GGGT		frameshift variant	-/GX	-	-	-	-	-	-
rs397516657	X:69616472	T/A		missense variant	L/Q	0.01	0.993	27	0.655	0.906	0.144
rs1131692034	X:69616488	C/A		stop gained	C/*	-	-	-	-	-	-
rs132630308	X:69616489	T/C		missense variant	Y/H	0	0.987	27	0.866	0.957	0.246
rs132630318	X:69616491	C/G		stop gained	Y/*	-	-	-	-	-	-
rs132630311	X:69616495	G/A		missense variant	E/K	0.01	0.587	28	0.833	0.923	0.18
rs132630319	X:69616501	C/G		missense variant	R/G	0.01	0.972	25	0.872	0.963	0.246
rs1057517971	X:69616553	G/-			G/X	-	-	-	-	-	-

				frameshift variant							
rs397516656	X: between 69616580 & 69616581	-/G		frameshift variant	S/RX	-	-	-	-	-	-
rs876657684	X: between 69616630 & 69616631	-/GGGC		frameshift variant	Q/RAX	-	-	-	-	-	-
rs397516660	X:69616637	C/A		stop gained	S/*	-	-	-	-	-	-
rs397516661	X:69616655	T/A		stop gained	L/*	-	-	-	-	-	-
rs727504537	X:69616705	G/A		splice donor variant	-	-	-	-	-	-	-
rs727504814	X:69616706	T/**C**/G		splice donor variant	-	-	-	-	-	-	-
rs727504814	X:69616706	T/C/**G**		splice donor variant	-	-	-	-	-	-	-
rs397516662	X:69957087	C/T		missense variant	R/C	**0.01**	**0.183**	**22**	**0.566**	**0.59**	**0.144**
rs132630312	X:69957093	C/T		missense variant	R/C	**0**	**0**	**22**	**0.452**	**0.548**	**0.144**
rs132630313	X:69957096	C/T		missense variant	R/C	**0**	**0.02**	**23**	**0.551**	**0.513**	**0.224**
rs132630314	X:69957097	G/**A**/T		missense variant	R/H	**0**	**0.865**	**25**	**0.54**	**0.754**	**0.224**

rs132630314	X:69957097	G/A/**T**		missense variant	R/L	0	0.394	23	0.518	0.668	0.224
rs727504649	X:69957104	A/C		missense variant	K/N	0	0.95	24	0.381	0.708	0.18
rs876657640	X:69957107	A/T		missense variant	R/S	0	0.879	25	0.381	0.705	0.088
rs1057521131	X:69957124	G/C		missense variant	G/A	0.11	0.995	23	0.493	0.751	0.557
rs727505013	X:69957133	G/A		splice donor variant	-	-	-	-	-	-	-
rs397516664	X:70023246	G/**C**/T		splice region variant intron variant	-	-	-	-	-	-	-
rs397516664	X:70023246	G/C/**T**		splice region variant intron variant	-	-	-	-	-	-	-
rs397516665	X:70027876-70027911	TGGACCCAATGGCCCTCC.../-		inframe deletion	PGPNGPPGPPGPP/P	-	-	-	-	-	-
rs397516666	X:70027883-70027918	AATGGCCCTCCAGGACCC.../-		inframe deletion	NGPPGPPGPPGP/-	-	-	-	-	-	-
rs397516667	X:70027892-70027919	CCAGGACCCCCAGGACCT.../-		frameshift variant	PGPPGPPGPQ/X	-	-	-	-	-	-
rs397516668	X:70027902-70027919	CAGGACCTCCAGGACCCC/-		inframe deletion	PGPPGPQ/Q	-	-	-	-	-	-

rs397516671	X:70027937	C/T		missense variant	P/S	0.02	0.999	26	0.527	0.873	0.492
rs1064793104	X:70027942-70027959	GATTCCTGGAATTCCAGG/-		inframe deletion	GIPGIPG/G	-	-	-	-	-	-
rs1057520742	X:70027947	C/T		missense variant	P/L	0.05	0.999	24	0.622	0.905	0.605
rs132630315	X:70027956	C/T	?	missense variant	P/L	0	0.999	24	0.735	0.883	0.704
rs876657685	X:70027978-70028013	ACCTGGTCCTCCAGGTCC... /-		inframe deletion	PPGPPGPPGPQGP/P	-	-	-	-	-	-
rs876657686	X:70027989-70028006	CAGGTCCTCCTGGTCCTC/-		inframe deletion	PGPPGPQ/Q	-	-	-	-	-	-
rs397516670	X:70027993-70028027	TCCTCCTGGTCCTCAAGG... /-		frameshift variant	GPPGPQGPPGLQG/GX	-	-	-	-	-	-
rs132630316	X:70028001	G/C		missense variant	G/A	0.02	0.999	27	0.978	0.989	0.893
rs727503007	X:70028006	C/T		stop gained	Q/*	-	-	-	-	-	-
rs886039344	X:70028037	G/A		splice donor variant	-	-	-	-	-	-	-
rs886039466	X:70029503	G/A		splice acceptor variant	-	-	-	-	-	-	-
rs397516672	X:70029527	C/T		stop gained	R/*	-	-	-	-	-	-

rs886042183	X:70029538	G/T	?	missense variant<br><br>splice region variant	Q/H	**0.01**	**0.967**	**29**	**0.469**	**0.75**	**0.331**
rs879255552	X:70030482	A/T		missense variant	H/L	**0**	**0.998**	**28**	**0.926**	**0.977**	**0.474**
rs1064793105	X:70030491	G/A		missense variant	G/D	**0**	**0.999**	**28**	**0.927**	**0.978**	**0.331**
rs727504417	X:70030493	C/**G**/T		missense variant	Q/E	**0.01**	**0.784**	**24**	**0.66**	**0.966**	**0.43**
rs727504417	X:70030493	C/G/**T**		stop gained	Q/*	-	-	-	-	-	-
rs1057517882	X:70030496	G/C		missense variant	G/R	**0.03**	**0.923**	**32**	**0.808**	**0.968**	**0.627**
rs879255611	X:70030503	C/A		missense variant	A/E	**0.01**	**0.754**	**25**	**0.815**	**0.955**	**0.43**
rs727503008	X:70033413	T/-		frameshift variant	V/X	-	-	-	-	-	-
rs397516675	X:70033426	G/**A**/T		stop gained	W/*	-	-	-	-	-	-
rs397516675	X:70033426	G/A/**T**		missense variant	W/C	**0**	**1**	**35**	**0.951**	**0.988**	**0.404**
rs397516676	X:70033426	G/-		frameshift variant	W/X	-	-	-	-	-	-

rs387907197	X:70033430	C/T		missense variant	R/C	0.01	0.968	28	0.817	0.953	0.065
rs1057517731	X:70033431	G/T		missense variant	R/L	0.05	0.329	26	0.697	0.901	0.065
rs879255551	X:70033469	C/T		missense variant	R/C	0	0.997	29	0.917	0.882	0.575
rs876657641	X:70033470	G/**A**/C		missense variant	R/H	0	0.992	34	0.821	0.881	0.389
rs876657641	X:70033470	G/A/**C**		missense variant	R/P	0	0.998	34	0.898	0.882	0.575
rs397516677	X:70033475	G/A		missense variant	G/R	0	1	33	0.948	0.985	0.675
rs886042021	X:70033476	G/A		missense variant	G/E	0	1	32	0.949	0.983	0.542
rs397516679	X:70033499	G/A		missense variant	G/S	0.01	1	28	0.958	0.996	0.81
rs397516681	X:70033506	A/G		missense variant	Y/C	0	0.999	29	0.969	0.977	0.799
rs876657642	X:70033515	A/C		missense variant	Y/S	0	0.998	29	0.968	0.945	0.81
rs727503009	X:70033521	A/**G**/T	?	missense variant	Q/R	0.01	0.986	27	0.918	0.987	0.808
rs727503009	X:70033521	A/G/**T**	?		Q/L	0	0.978	28	0.955	0.987	0.808

				missense variant							
rs1131691566	X:70033526	G/T		stop gained<br><br>splice region variant	E/*	-	-	-	-	-	-
rs1064793106	X:70033527	A/G		missense variant<br><br>splice region variant	E/G	**0**	**1**	**35**	**0.758**	**0.842**	**0.297**
rs1085307599	X:70035364	T/C		missense variant	Y/H	**0**	**0.966**	**27**	**0.927**	**0.978**	**0.566**
rs1057518211	X:70035365	A/C		missense variant	Y/S	**0**	**0.786**	**30**	**0.929**	**0.981**	**0.694**
rs727503010	X:70035381	C/-		frameshift variant	D/X	-	-	-	-	-	-
rs876657687	X:70035393	T/**C**/G		synonymous variant	Y	-	-	-	-	-	-
rs876657687	X:70035393	T/C/**G**		stop gained	Y/*	-	-	-	-	-	-
rs397516682	X:70035394	G/T		stop gained	E/*	-	-	-	-	-	-
rs727503011	X:70035424	C/T		stop gained	Q/*	-	-	-	-	-	-
rs142948132	X:70035434	G/**A**/C/T		missense variant	R/H	**0.06**	**0.951**	**27**	**0.769**	**0.792**	**0.31**

rs142948132	X:70035434	G/A/**C**/T	?	missense variant	R/P	0.27	0.974	27	0.843	0.863	0.399
rs142948132	X:70035434	G/A/C/**T**	?	missense variant	R/L	0.13	0.832	27	0.788	0.812	0.31
rs132630321	X:70035446	C/T		missense variant	T/M	0	0.954	24	0.875	0.873	0.519
rs132630317	X:70035478	G/**A**/T		missense variant	A/T	0	0.997	26	0.887	0.981	0.687
rs132630317	X:70035478	G/A/**T**		missense variant	A/S	0.12	0.996	24	0.726	0.979	0.482
rs876657639	X:70035500	C/T		missense variant	A/V	0.01	0.99	27	0.833	0.872	0.331
rs886039347	X:70035502	C/T	?	missense variant	R/W	0.01	0.915	27	0.749	0.948	0.17
rs132630320	X:70035505	C/G		missense variant	Q/E	0.02	0.969	24	0.783	0.959	0.18
rs397516654	X:70035527	T/C		missense variant	V/A	0.07	0.598	24	0.666	0.926	0.119
rs781394318	X:70035556	A/G		missense variant	K/E	0.02	0.041	25	0.591	0.824	0.028
rs780582849	X:70035570	C/**A**/T		missense variant	F/L	0	0.967	26	0.938	0.934	0.784
rs780582849	X:70035570	C/A/**T**			F	-	-	-	-	-	-

				synonymous variant							
rs749830948	X:70035577	G/**A**/T		missense variant	A/T	0.02	0.652	26	0.771	0.831	0.298
rs749830948	X:70035577	G/A/**T**		missense variant	A/S	0.54	0.186	22	0.479	0.701	0.104
rs397516659	X:69616310	T/C		start lost	M/T	0	0.122	22	0.689	0.919	-
rs132630310	X:69616375	C/T		stop gained	Q/*	-	-	-	-	-	-
rs727505089	X: between 69616443 & 69616444	-/GGGT		frameshift variant	-/GX	-	-	-	-	-	-
rs397516657	X:69616472	T/A		missense variant	L/Q	0.01	0.997	27	0.655	0.906	0.144
rs1131692034	X:69616488	C/A		stop gained	C/*	-	-	-	-	-	-
rs132630308	X:69616489	T/C		missense variant	Y/H	0	0.994	27	0.866	0.957	0.246
rs132630318	X:69616491	C/G		stop gained	Y/*	-	-	-	-	-	-
rs132630311	X:69616495	G/A		missense variant	E/K	0.01	0.765	28	0.833	0.923	0.18
rs132630319	X:69616501	C/G		missense variant	R/G	0.01	0.988	25	0.872	0.963	0.246
rs1057517971	X:69616553	G/-		frameshift variant	G/X	-	-	-	-	-	-

rs397516656	X: between 69616580 & 69616581	-/G		frameshift variant	S/RX	-	-	-	-	-	-
rs876657684	X: between 69616630 & 69616631	-/GGGC		frameshift variant	Q/RAX	-	-	-	-	-	-
rs397516660	X:69616637	C/A		stop gained	S/*	-	-	-	-	-	-
rs397516661	X:69616655	T/A		stop gained	L/*	-	-	-	-	-	-
rs727504537	X:69616705	G/A		splice donor variant	-	-	-	-	-	-	-
rs727504814	X:69616706	T/**C**/G		splice donor variant	-	-	-	-	-	-	-
rs727504814	X:69616706	T/C/**G**		splice donor variant	-	-	-	-	-	-	-
rs397516662	X:69957087	C/T		missense variant	R/C	**0.01**	**0.34**	**22**	**0.566**	**0.59**	**0.144**
rs132630312	X:69957093	C/T		missense variant	R/C	**0**	**0.003**	**22**	**0.452**	**0.548**	**0.144**
rs132630313	X:69957096	C/T		missense variant	R/C	**0**	**0.044**	**23**	**0.551**	**0.513**	**0.224**
rs132630314	X:69957097	G/**A**/T		missense variant	R/H	**0**	**0.936**	**25**	**0.54**	**0.754**	**0.224**
rs132630314	X:69957097	G/A/**T**		missense variant	R/L	**0**	**0.599**	**23**	**0.518**	**0.668**	**0.224**

rs727504649	X:69957104	A/C		missense variant	K/N	**0**	**0.978**	**24**	**0.381**	**0.708**	**0.18**
rs876657640	X:69957107	A/T		missense variant	R/S	**0**	**0.943**	**25**	**0.381**	**0.705**	**0.088**
rs1057521131	X:69957124	G/C		missense variant	G/A	**0.13**	**0.992**	**23**	**0.493**	**0.751**	**0.557**
rs727505013	X:69957133	G/A		splice donor variant	-	-	-	-	-	-	-
rs397516664	X:70023246	G/**C**/T		splice region variant intron variant	-	-	-	-	-	-	-
rs397516664	X:70023246	G/C/**T**		splice region variant intron variant	-	-	-	-	-	-	-
rs397516665	X:70027876-70027911	TGGACCCAATGGCCCTCC... /-		inframe deletion	PGPNGPPGPPGPP/P	-	-	-	-	-	-
rs397516666	X:70027883-70027918	AATGGCCCTCCAGGACCC.. ./-		inframe deletion	NGPPGPPGPPGP/-	-	-	-	-	-	-
rs397516667	X:70027892-70027919	CCAGGACCCCCAGGACCT.. ./-		frameshift variant	PGPPGPPGPQ/X	-	-	-	-	-	-
rs397516668	X:70027902-70027919	CAGGACCTCCAGGACCCC/-		inframe deletion	PGPPGPQ/Q	-	-	-	-	-	-
rs397516671	X:70027937	C/T		missense variant	P/S	**0.02**	**0.998**	**26**	**0.527**	**0.873**	**0.492**

rs1064793104	X:70027942-70027959	GATTCCTGGAATTCCAGG/-		inframe deletion	GIPGIPG/G	-	-	-	-	-	-
rs1057520742	X:70027947	C/T		missense variant	P/L	0.04	0.998	24	0.622	0.905	0.605
rs132630315	X:70027956	C/T	?	missense variant	P/L	0	0.998	24	0.735	0.883	0.704
rs876657685	X:70027978-70028013	ACCTGGTCCTCCAGGTCC... /-		inframe deletion	PPGPPGPPGPQGP/P	-	-	-	-	-	-
rs876657686	X:70027989-70028006	CAGGTCCTCCTGGTCCTC/-		inframe deletion	PGPPGPQ/Q	-	-	-	-	-	-
rs397516670	X:70027993-70028027	TCCTCCTGGTCCTCAAGG... /-		frameshift variant	GPPGPQGPPGLQG/GX	-	-	-	-	-	-
rs132630316	X:70028001	G/C		missense variant	G/A	0.05	0.998	27	0.978	0.989	0.893
rs727503007	X:70028006	C/T		stop gained	Q/*	-	-	-	-	-	-
rs886039344	X:70028037	G/A		splice donor variant	-	-	-	-	-	-	-
rs886039466	X:70029503	G/A		splice acceptor variant	-	-	-	-	-	-	-
rs397516672	X:70029527	C/T		stop gained	R/*	-	-	-	-	-	-
rs886042183	X:70029538	G/T	?	missense variant	Q/H	0.01	0.944	29	0.469	0.75	0.331

				splice region variant							
rs879255552	X:70030482	A/T		missense variant	H/L	0	0.999	28	0.926	0.977	0.474
rs1064793105	X:70030491	G/A		missense variant	G/D	0	1	28	0.927	0.978	0.331
rs727504417	X:70030493	C/**G**/T		missense variant	Q/E	0.01	0.893	24	0.66	0.966	0.43
rs727504417	X:70030493	C/G/**T**		stop gained	Q/*	-	-	-	-	-	-
rs1057517882	X:70030496	G/C		missense variant	G/R	0.04	0.965	32	0.808	0.968	0.627
rs879255611	X:70030503	C/A		missense variant	A/E	0.01	0.876	25	0.815	0.955	0.43
rs727503008	X:70033413	T/-		frameshift variant	V/X	-	-	-	-	-	-
rs397516676	X:70033426	G/-		frameshift variant	W/X	-	-	-	-	-	-
rs397516675	X:70033426	G/**A**/T		stop gained	W/*	-	-	-	-	-	-
rs397516675	X:70033426	G/A/**T**		missense variant	W/C	0	0.999	35	0.951	0.988	0.404
rs387907197	X:70033430	C/T		missense variant	R/C	0.01	0.945	28	0.817	0.953	0.065

rs1057517731	X:70033431	G/T		missense variant	R/L	**0.05**	**0.221**	**26**	**0.697**	**0.901**	**0.065**
rs879255551	X:70033469	C/T		missense variant	R/C	**0**	**0.995**	**29**	**0.917**	**0.882**	**0.575**
rs876657641	X:70033470	G/**A**/C		missense variant	R/H	**0**	**0.986**	**34**	**0.821**	**0.881**	**0.389**
rs876657641	X:70033470	G/A/**C**		missense variant	R/P	**0**	**0.996**	**34**	**0.898**	**0.882**	**0.575**
rs397516677	X:70033475	G/A		missense variant	G/R	**0**	**1**	**33**	**0.948**	**0.985**	**0.675**
rs886042021	X:70033476	G/A		missense variant	G/E	**0**	**1**	**32**	**0.949**	**0.983**	**0.542**
rs397516679	X:70033499	G/A		missense variant	G/S	**0**	**0.999**	**28**	**0.958**	**0.996**	**0.81**
rs397516681	X:70033506	A/G		missense variant	Y/C	**0**	**0.998**	**29**	**0.969**	**0.977**	**0.799**
rs876657642	X:70033515	A/C		missense variant	Y/S	**0**	**0.997**	**29**	**0.968**	**0.945**	**0.81**
rs727503009	X:70033521	A/**G**/T	?	missense variant<br><br>splice region variant	Q/R	**0**	**0.975**	**27**	**0.918**	**0.987**	**0.808**
rs727503009	X:70033521	A/G/**T**	?	missense variant	Q/L	**0**	**0.962**	**28**	**0.955**	**0.987**	**0.808**

				splice region variant							
rs1131691566	X:70033526	G/T		splice region variant intron variant	-	-	-	-	-	-	-
rs1064793106	X:70033527	A/G		splice region variant intron variant	-	-	-	-	-	-	-
rs1085307599	X:70035364	T/C		missense variant	Y/H	**0**	**0.996**	**27**	**0.927**	**0.978**	**0.566**
rs1057518211	X:70035365	A/C		missense variant	Y/S	**0**	**0.994**	**30**	**0.929**	**0.981**	**0.694**
rs727503010	X:70035381	C/-		frameshift variant	D/X	-	-	-	-	-	-
rs876657687	X:70035393	T/**C**/G		synonymous variant	Y	-	-	-	-	-	-
rs876657687	X:70035393	T/C/**G**		stop gained	Y/*	-	-	-	-	-	-
rs397516682	X:70035394	G/T		stop gained	E/*	-	-	-	-	-	-
rs727503011	X:70035424	C/T		stop gained	Q/*	-	-	-	-	-	-
rs142948132	X:70035434	G/**A**/C/T		missense variant	R/H	**0.06**	**0.919**	**27**	**0.769**	**0.792**	**0.31**
rs142948132	X:70035434	G/A/**C**/T		missense variant	R/P	**0.27**	**0.956**	**27**	**0.843**	**0.863**	**0.399**

rs142948132	X:70035434	G/A/C/**T**	?	missense variant	R/L	0.13	0.741	27	0.788	0.812	0.31
rs132630321	X:70035446	C/T		missense variant	T/M	0	0.923	24	0.875	0.873	0.519
rs132630317	X:70035478	G/**A**/T		missense variant	A/T	0	0.989	26	0.887	0.981	0.687
rs132630317	X:70035478	G/A/**T**		missense variant	A/S	0.13	0.994	24	0.726	0.979	0.482
rs876657639	X:70035500	C/T		missense variant	A/V	0.01	0.982	27	0.833	0.872	0.331
rs886039347	X:70035502	C/T	?	missense variant	R/W	0.01	0.861	27	0.749	0.948	0.17
rs132630320	X:70035505	C/G		missense variant	Q/E	0.02	0.948	24	0.783	0.959	0.18
rs397516654	X:70035527	T/C		missense variant	V/A	0.1	0.463	24	0.666	0.926	0.119
rs781394318	X:70035556	A/G		missense variant	K/E	0.02	0.079	25	0.591	0.824	0.028
rs780582849	X:70035570	C/**A**/T		missense variant	F/L	0	0.962	26	0.938	0.934	0.784
rs780582849	X:70035570	C/A/**T**		synonymous variant	F	-	-	-	-	-	-
rs749830948	X:70035577	G/**A**/T			A/T	0.02	0.642	26	0.771	0.831	0.298

				missense variant							
rs749830948	X:70035577	G/A/**T**		missense variant	A/S	**0.58**	**0.185**	**22**	**0.479**	**0.701**	**0.104**
rs397516659	X:69616310	T/C		non coding transcript exon variant	-	-	-	-	-	-	-
rs132630310	X:69616375	C/T		non coding transcript exon variant	-	-	-	-	-	-	-
rs727505089	X: between 69616443 & 69616444	-/GGGT		non coding transcript exon variant	-	-	-	-	-	-	-
rs397516657	X:69616472	T/A		non coding transcript exon variant	-	-	-	-	-	-	-
rs1131692034	X:69616488	C/A		non coding transcript exon variant	-	-	-	-	-	-	-
rs132630308	X:69616489	T/C		non coding transcript exon variant	-	-	-	-	-	-	-
rs132630318	X:69616491	C/G		non coding transcript exon variant	-	-	-	-	-	-	-
rs132630311	X:69616495	G/A		non coding transcript exon variant	-	-	-	-	-	-	-
rs132630319	X:69616501	C/G		non coding transcript exon variant	-	-	-	-	-	-	-
rs1057517971	X:69616553	G/-		non coding transcript exon variant	-	-	-	-	-	-	-

rs397516656	X: between 69616580 & 69616581	-/G		non coding transcript exon variant	-	-	-	-	-	-	-	-
rs876657684	X: between 69616630 & 69616631	-/GGGC		non coding transcript exon variant	-	-	-	-	-	-	-	-
rs397516660	X:69616637	C/A		non coding transcript exon variant	-	-	-	-	-	-	-	-
rs397516661	X:69616655	T/A		non coding transcript exon variant	-	-	-	-	-	-	-	-
rs727504537	X:69616705	G/A		splice donor variant non coding transcript variant	-	-	-	-	-	-	-	-
rs727504814	X:69616706	T/**C**/G		splice donor variant non coding transcript variant	-	-	-	-	-	-	-	-
rs727504814	X:69616706	T/C/**G**		splice donor variant non coding transcript variant	-	-	-	-	-	-	-	-
rs397516662	X:69957087	C/T		non coding transcript exon variant	-	-	-	-	-	-	-	-
rs132630312	X:69957093	C/T		non coding transcript exon variant	-	-	-	-	-	-	-	-

rs132630313	X:69957096	C/T		non coding transcript exon variant	-	-	-	-	-	-	-
rs132630314	X:69957097	G/**A**/T		non coding transcript exon variant	-	-	-	-	-	-	-
rs132630314	X:69957097	G/A/**T**		non coding transcript exon variant	-	-	-	-	-	-	-
rs727504649	X:69957104	A/C		non coding transcript exon variant	-	-	-	-	-	-	-
rs876657640	X:69957107	A/T		non coding transcript exon variant	-	-	-	-	-	-	-
rs1057521131	X:69957124	G/C		non coding transcript exon variant	-	-	-	-	-	-	-
rs727505013	X:69957133	G/A		non coding transcript exon variant	-	-	-	-	-	-	-
rs397516662	X:69957087	C/T		missense variant	R/C	**0**	**0.183**	**22**	**0.566**	**0.59**	**0.144**
rs132630312	X:69957093	C/T		missense variant	R/C	**0**	**0**	**22**	**0.452**	**0.548**	**0.144**
rs132630313	X:69957096	C/T		missense variant	R/C	**0**	**0.02**	**23**	**0.551**	**0.513**	**0.224**
rs132630314	X:69957097	G/**A**/T		missense variant	R/H	**0.01**	**0.865**	**25**	**0.54**	**0.754**	**0.224**
rs132630314	X:69957097	G/A/**T**			R/L	**0.17**	**0.394**	**23**	**0.518**	**0.668**	**0.224**

				missense variant							
rs727504649	X:69957104	A/C		missense variant	K/N	**0.01**	**0.95**	**24**	**0.381**	**0.708**	**0.18**
rs876657640	X:69957107	A/T		missense variant	R/S	**0**	**0.879**	**25**	**0.381**	**0.705**	**0.088**
rs1057521131	X:69957124	G/C		missense variant	G/A	**0.12**	**0.995**	**23**	**0.493**	**0.751**	**0.557**
rs727505013	X:69957133	G/A		splice donor variant	-	-	-	-	-	-	-
rs397516664	X:70023246	G/**C**/T		splice region variant intron variant	-	-	-	-	-	-	-
rs397516664	X:70023246	G/C/**T**		splice region variant intron variant	-	-	-	-	-	-	-
rs397516665	X:70027876-70027911	TGGACCCAATGGCCCTCC.../-		inframe deletion	PGPNGPPGPPGPP/P	-	-	-	-	-	-
rs397516666	X:70027883-70027918	AATGGCCCTCCAGGACCC.../-		inframe deletion	NGPPGPPGPPGP/-	-	-	-	-	-	-
rs397516667	X:70027892-70027919	CCAGGACCCCCAGGACCT.../-		frameshift variant	PGPPGPPGPQ/X	-	-	-	-	-	-
rs397516668	X:70027902-70027919	CAGGACCTCCAGGACCCC/-		inframe deletion	PGPPGPQ/Q	-	-	-	-	-	-

rs397516671	X:70027937	C/T		missense variant	P/S	0	0.999	26	0.527	0.873	0.492
rs1064793104	X:70027942-70027959	GATTCCTGGAATTCCAGG/-		inframe deletion	GIPGIPG/G	-	-	-	-	-	-
rs1057520742	X:70027947	C/T		missense variant	P/L	0.01	0.999	24	0.622	0.905	0.605
rs132630315	X:70027956	C/T		missense variant	P/L	0	0.999	24	0.735	0.883	0.704
rs876657685	X:70027978-70028013	ACCTGGTCCTCCAGGTCC... /-		inframe deletion	PPGPPGPPGPQGP/P	-	-	-	-	-	-
rs876657686	X:70027989-70028006	CAGGTCCTCCTGGTCCTC/-		inframe deletion	PGPPGPQ/Q	-	-	-	-	-	-
rs397516670	X:70027993-70028027	TCCTCCTGGTCCTCAAGG... /-		frameshift variant	GPPGPQGPPGLQG/GX	-	-	-	-	-	-
rs132630316	X:70028001	G/C		missense variant	G/A	0	0.999	27	0.978	0.989	0.893
rs727503007	X:70028006	C/T		stop gained	Q/*	-	-	-	-	-	-
rs886039344	X:70028037	G/A		splice donor variant	-	-	-	-	-	-	-
rs886039466	X:70029503	G/A		splice acceptor variant	-	-	-	-	-	-	-
rs397516672	X:70029527	C/T		stop gained	R/*	-	-	-	-	-	-

rs886042183	X:70029538	G/T	?	missense variant<br><br>splice region variant	Q/H	0	0.967	29	0.469	0.75	0.331
rs879255552	X:70030482	A/T		missense variant	H/L	0	0.998	28	0.926	0.977	0.474
rs1064793105	X:70030491	G/A		missense variant	G/D	0	0.999	28	0.927	0.978	0.331
rs727504417	X:70030493	C/**G**/T		missense variant	Q/E	0	0.784	24	0.66	0.966	0.43
rs727504417	X:70030493	C/G/**T**		stop gained	Q/*	-	-	-	-	-	-
rs1057517882	X:70030496	G/C		missense variant	G/R	0	0.923	32	0.808	0.968	0.627
rs879255611	X:70030503	C/A		missense variant	A/E	0	0.754	25	0.815	0.955	0.43
rs397516659	X:69616310	T/C		start lost	M/T	0	0.122	22	0.689	0.919	-
rs132630310	X:69616375	C/T		stop gained	Q/*	-	-	-	-	-	-
rs727505089	X: between 69616443 & 69616444	-/GGGT		frameshift variant	-/GX	-	-	-	-	-	-
rs397516657	X:69616472	T/A		missense variant	L/Q	0.01	0.997	27	0.655	0.906	0.144
rs1131692034	X:69616488	C/A		stop gained	C/*	-	-	-	-	-	-

rs132630308	X:69616489	T/C		missense variant	Y/H	**0**	**0.994**	**27**	**0.866**	**0.957**	**0.246**
rs132630318	X:69616491	C/G		stop gained	Y/*	-	-	-	-	-	-
rs132630311	X:69616495	G/A		missense variant	E/K	**0.01**	**0.765**	**28**	**0.833**	**0.923**	**0.18**
rs132630319	X:69616501	C/G		missense variant	R/G	**0.01**	**0.988**	**25**	**0.872**	**0.963**	**0.246**
rs1057517971	X:69616553	G/-		frameshift variant	G/X	-	-	-	-	-	-
rs397516656	X: between 69616580 & 69616581	-/G		frameshift variant	S/RX	-	-	-	-	-	-
rs876657684	X: between 69616630 & 69616631	-/GGGC		frameshift variant	Q/RAX	-	-	-	-	-	-
rs397516660	X:69616637	C/A		stop gained	S/*	-	-	-	-	-	-
rs397516661	X:69616655	T/A		stop gained	L/*	-	-	-	-	-	-
rs727504537	X:69616705	G/A		splice donor variant	-	-	-	-	-	-	-
rs727504814	X:69616706	T/**C**/G		splice donor variant	-	-	-	-	-	-	-
rs727504814	X:69616706	T/C/**G**		splice donor variant	-	-	-	-	-	-	-

rs397516662	X:69957087	C/T		missense variant	R/C	0.02	0.34	22	0.566	0.59	0.144
rs132630312	X:69957093	C/T		missense variant	R/C	0	0.003	22	0.452	0.548	0.144
rs132630313	X:69957096	C/T		missense variant	R/C	0	0.044	23	0.551	0.513	0.224
rs132630314	X:69957097	G/**A**/T		missense variant	R/H	0	0.936	25	0.54	0.754	0.224
rs132630314	X:69957097	G/A/**T**		missense variant	R/L	0	0.599	23	0.518	0.668	0.224
rs727504649	X:69957104	A/C		missense variant	K/N	0	0.978	24	0.381	0.708	0.18
rs876657640	X:69957107	A/T		missense variant	R/S	0.01	0.943	25	0.381	0.705	0.088
rs1057521131	X:69957124	G/C		missense variant	G/A	0.11	0.992	23	0.493	0.751	0.557
rs727505013	X:69957133	G/A		splice donor variant	-	-	-	-	-	-	-
rs397516664	X:70023246	G/**C**/T		splice region variant intron variant	-	-	-	-	-	-	-
rs397516664	X:70023246	G/C/**T**		splice region variant intron variant	-	-	-	-	-	-	-

rs397516665	X:70027876-70027911	TGGACCCAATGGCCCTCC.../-		inframe deletion	PGPNGPPGPPGPP/P	-	-	-	-	-	-
rs397516666	X:70027883-70027918	AATGGCCCTCCAGGACCC../-		inframe deletion	NGPPGPPGPPGP/-	-	-	-	-	-	-
rs397516667	X:70027892-70027919	CCAGGACCCCCAGGACCT../-		frameshift variant	PGPPGPPGPQ/X	-	-	-	-	-	-
rs397516668	X:70027902-70027919	CAGGACCTCCAGGACCCC/-		inframe deletion	PGPPGPQ/Q	-	-	-	-	-	-
rs397516671	X:70027937	C/T		missense variant	P/S	**0.01**	**0.998**	**26**	**0.527**	**0.873**	**0.492**
rs1064793104	X:70027942-70027959	GATTCCTGGAATTCCAGG/-		inframe deletion	GIPGIPG/G	-	-	-	-	-	-
rs1057520742	X:70027947	C/T		missense variant	P/L	**0.04**	**0.998**	**24**	**0.622**	**0.905**	**0.605**
rs132630315	X:70027956	C/T	?	missense variant	P/L	**0.01**	**0.998**	**24**	**0.735**	**0.883**	**0.704**
rs876657685	X:70027978-70028013	ACCTGGTCCTCCAGGTCC.../-		inframe deletion	PPGPPGPPGPQGP/P	-	-	-	-	-	-
rs876657686	X:70027989-70028006	CAGGTCCTCCTGGTCCTC/-		inframe deletion	PGPPGPQ/Q	-	-	-	-	-	-
rs397516670	X:70027993-70028027	TCCTCCTGGTCCTCAAGG.../-		frameshift variant	GPPGPQGPPGLQG/GX	-	-	-	-	-	-
rs132630316	X:70028001	G/C			G/A	**0.03**	**0.998**	**27**	**0.978**	**0.989**	**0.893**

				missense variant							
rs727503007	X:70028006	C/T		stop gained	Q/*	-	-	-	-	-	-
rs886039344	X:70028037	G/A		splice donor variant	-	-	-	-	-	-	-
rs886039466	X:70029503	G/A		splice acceptor variant	-	-	-	-	-	-	-
rs397516672	X:70029527	C/T		stop gained	R/*	-	-	-	-	-	-
rs886042183	X:70029538	G/T	?	missense variant<br><br>splice region variant	Q/H	0.01	0.944	29	0.469	0.75	0.331
rs879255552	X:70030482	A/T		missense variant	H/L	0	0.999	28	0.926	0.977	0.474
rs1064793105	X:70030491	G/A		missense variant	G/D	0	1	28	0.927	0.978	0.331
rs727504417	X:70030493	C/**G**/T		missense variant	Q/E	0.02	0.893	24	0.66	0.966	0.43
rs727504417	X:70030493	C/G/**T**		stop gained	Q/*	-	-	-	-	-	-
rs1057517882	X:70030496	G/C		missense variant	G/R	0.15	0.965	32	0.808	0.968	0.627
rs879255611	X:70030503	C/A			A/E	0.06	0.876	25	0.815	0.955	0.43

				missense variant							
rs727503008	X:70033413	T/-		frameshift variant	V/X	-	-	-	-	-	-
rs397516675	X:70033426	G/**A**/T		stop gained	W/*	-	-	-	-	-	-
rs397516675	X:70033426	G/A/**T**		missense variant	W/C	0	0.999	35	0.951	0.988	0.404
rs397516676	X:70033426	G/-		frameshift variant	W/X	-	-	-	-	-	-
rs387907197	X:70033430	C/T		missense variant	R/C	0	0.945	28	0.817	0.953	0.065
rs1057517731	X:70033431	G/T		missense variant	R/L	0.02	0.221	26	0.697	0.901	0.065
rs879255551	X:70033469	C/T		missense variant	R/C	0	0.995	29	0.917	0.882	0.575
rs876657641	X:70033470	G/**A**/C		missense variant	R/H	0.08	0.986	34	0.821	0.881	0.389
rs876657641	X:70033470	G/A/**C**		missense variant	R/P	0	0.996	34	0.898	0.882	0.575
rs397516677	X:70033475	G/A		missense variant	G/R	0	1	33	0.948	0.985	0.675
rs886042021	X:70033476	G/A		missense variant	G/E	0	1	32	0.949	0.983	0.542

rs397516679	X:70033499	G/A		missense variant	G/S	0	0.999	28	0.958	0.996	0.81
rs397516681	X:70033506	A/G		missense variant	Y/C	0	0.998	29	0.969	0.977	0.799
rs876657642	X:70033515	A/C		missense variant	Y/S	0	0.997	29	0.968	0.945	0.81
rs727503009	X:70033521	A/**G**/T	?	missense variant<br><br>splice region variant	Q/R	0	0.975	27	0.918	0.987	0.808
rs727503009	X:70033521	A/G/**T**	?	missense variant<br><br>splice region variant	Q/L	0	0.962	28	0.955	0.987	0.808
rs1131691566	X:70033526	G/T		splice region variant intron variant	-	-	-	-	-	-	-
rs1064793106	X:70033527	A/G		splice region variant intron variant	-	-	-	-	-	-	-
rs1085307599	X:70035364	T/C		missense variant	Y/H	0	0.983	27	0.927	0.978	0.566
rs1057518211	X:70035365	A/C		missense variant	Y/S	0	0.958	30	0.929	0.981	0.694
rs727503010	X:70035381	C/-			D/X	-	-	-	-	-	-

				frameshift variant							
rs876657687	X:70035393	T/**C**/G		synonymous variant	Y	-	-	-	-	-	-
rs876657687	X:70035393	T/C/**G**		stop gained	Y/*	-	-	-	-	-	-
rs397516682	X:70035394	G/T		stop gained	E/*	-	-	-	-	-	-
rs727503011	X:70035424	C/T		stop gained	Q/*	-	-	-	-	-	-
rs142948132	X:70035434	G/**A**/C/T		missense variant	R/H	**0.06**	**0.919**	**27**	**0.769**	**0.792**	**0.31**
rs142948132	X:70035434	G/A/**C**/T		missense variant	R/P	**0.27**	**0.956**	**27**	**0.843**	**0.863**	**0.399**
rs142948132	X:70035434	G/A/C/**T**		missense variant	R/L	**0.13**	**0.741**	**27**	**0.788**	**0.812**	**0.31**
rs132630321	X:70035446	C/T		missense variant	T/M	**0**	**0.923**	**24**	**0.875**	**0.873**	**0.519**
rs132630317	X:70035478	G/**A**/T		missense variant	A/T	**0**	**0.989**	**26**	**0.887**	**0.981**	**0.687**
rs132630317	X:70035478	G/A/**T**		missense variant	A/S	**0.19**	**0.994**	**24**	**0.726**	**0.979**	**0.482**
rs876657639	X:70035500	C/T		missense variant	A/V	**0.01**	**0.982**	**27**	**0.833**	**0.872**	**0.331**
rs886039347	X:70035502	C/T			R/W	**0.01**	**0.861**	**27**	**0.749**	**0.948**	**0.17**

				missense variant							
rs132630320	X:70035505	C/G		missense variant	Q/E	0.02	0.948	24	0.783	0.959	0.18
rs397516654	X:70035527	T/C		missense variant	V/A	0.12	0.463	24	0.666	0.926	0.119
rs781394318	X:70035556	A/G		missense variant	K/E	0.05	0.107	25	0.591	0.824	0.028
rs780582849	X:70035570	C/**A**/T		missense variant	F/L	0	0.962	26	0.938	0.934	0.784
rs780582849	X:70035570	C/A/**T**		synonymous variant	F	-	-	-	-	-	-
rs749830948	X:70035577	G/**A**/T		missense variant	A/T	0.03	0.642	26	0.771	0.831	0.298
rs749830948	X:70035577	G/A/**T**		missense variant	A/S	0.57	0.185	22	0.479	0.701	0.104
rs397516659	X:69616310	T/C		start lost	M/T	0	0.733	22	0.689	0.919	-
rs132630310	X:69616375	C/T		stop gained	Q/*	-	-	-	-	-	-
rs727505089	X: between 69616443 & 69616444	-/GGGT		frameshift variant	-/GX	-	-	-	-	-	-
rs397516657	X:69616472	T/A		missense variant	L/Q	0	0.999	27	0.655	0.906	0.144

rs1131692034	X:69616488	C/A		stop gained	C/*	-	-	-	-	-	-
rs132630308	X:69616489	T/C		missense variant	Y/H	**0**	**0.998**	**27**	**0.866**	**0.957**	**0.246**
rs132630318	X:69616491	C/G		stop gained	Y/*	-	-	-	-	-	-
rs132630311	X:69616495	G/A		missense variant	E/K	**0.01**	**0.957**	**28**	**0.833**	**0.923**	**0.18**
rs132630319	X:69616501	C/G		missense variant	R/G	**0**	**0.998**	**25**	**0.872**	**0.963**	**0.246**
rs1057517971	X:69616553	G/-		frameshift variant	G/X	-	-	-	-	-	-
rs397516656	X: between 69616580 & 69616581	-/G		frameshift variant	S/RX	-	-	-	-	-	-
rs876657684	X: between 69616630 & 69616631	-/GGGC		frameshift variant	Q/RAX	-	-	-	-	-	-
rs397516660	X:69616637	C/A		stop gained	S/*	-	-	-	-	-	-
rs397516661	X:69616655	T/A		stop gained	L/*	-	-	-	-	-	-
rs727504537	X:69616705	G/A		splice donor variant	-	-	-	-	-	-	-
rs727504814	X:69616706	T/**C**/G		splice donor variant	-	-	-	-	-	-	-
rs727504814	X:69616706	T/C/**G**			-	-	-	-	-	-	-

				splice donor variant							
rs397516659	X:69616310	T/C		start lost	M/T	0	0.806	22	0.689	0.919	-
rs132630310	X:69616375	C/T		stop gained	Q/*	-	-	-	-	-	-
rs727505089	X: between 69616443 & 69616444	-/GGGT		frameshift variant	-/GX	-	-	-	-	-	-
rs397516657	X:69616472	T/A		missense variant	L/Q	0	0.999	27	0.655	0.906	0.144
rs1131692034	X:69616488	C/A		stop gained	C/*	-	-	-	-	-	-
rs132630308	X:69616489	T/C		missense variant	Y/H	0	0.998	27	0.866	0.957	0.246
rs132630318	X:69616491	C/G		stop gained	Y/*	-	-	-	-	-	-
rs132630311	X:69616495	G/A		missense variant	E/K	0.01	0.971	28	0.833	0.923	0.18
rs132630319	X:69616501	C/G		missense variant	R/G	0	0.999	25	0.872	0.963	0.246
rs1057517971	X:69616553	G/-		frameshift variant	G/X	-	-	-	-	-	-
rs397516656	X: between 69616580 & 69616581	-/G		frameshift variant	S/RX	-	-	-	-	-	-
rs876657684	X: between 69616630 & 69616631	-/GGGC		frameshift variant	Q/RAX	-	-	-	-	-	-

rs397516660	X:69616637	C/A		stop gained	S/*	-	-	-	-	-	-
rs397516661	X:69616655	T/A		stop gained	L/*	-	-	-	-	-	-
rs727504537	X:69616705	G/A		splice donor variant	-	-	-	-	-	-	-
rs727504814	X:69616706	T/**C**/G		splice donor variant	-	-	-	-	-	-	-
rs727504814	X:69616706	T/C/**G**		splice donor variant	-	-	-	-	-	-	-
rs397516659	X:69616310	T/C		non coding transcript exon variant	-	-	-	-	-	-	-
rs132630310	X:69616375	C/T		non coding transcript exon variant	-	-	-	-	-	-	-
rs727505089	X: between 69616443 & 69616444	-/GGGT		non coding transcript exon variant	-	-	-	-	-	-	-
rs397516657	X:69616472	T/A		non coding transcript exon variant	-	-	-	-	-	-	-
rs1131692034	X:69616488	C/A		non coding transcript exon variant	-	-	-	-	-	-	-
rs132630308	X:69616489	T/C		non coding transcript exon variant	-	-	-	-	-	-	-
rs132630318	X:69616491	C/G		non coding transcript exon variant	-	-	-	-	-	-	-

rs132630311	X:69616495	G/A		non coding transcript exon variant	-	-	-	-	-	-	-
rs132630319	X:69616501	C/G		non coding transcript exon variant	-	-	-	-	-	-	-
rs1057517971	X:69616553	G/-		non coding transcript exon variant	-	-	-	-	-	-	-
rs397516656	X: between 69616580 & 69616581	-/G		non coding transcript exon variant	-	-	-	-	-	-	-
rs876657684	X: between 69616630 & 69616631	-/GGGC		non coding transcript exon variant	-	-	-	-	-	-	-
rs397516660	X:69616637	C/A		non coding transcript exon variant	-	-	-	-	-	-	-
rs397516661	X:69616655	T/A		non coding transcript exon variant	-	-	-	-	-	-	-
rs727504537	X:69616705	G/A		splice donor variant non coding transcript variant	-	-	-	-	-	-	-
rs727504814	X:69616706	T/**C**/G		splice donor variant non coding transcript variant	-	-	-	-	-	-	-
rs727504814	X:69616706	T/C/**G**		splice donor variant	-	-	-	-	-	-	-

				non coding transcript variant							
rs397516662	X:69957087	C/T		non coding transcript exon variant	-	-	-	-	-	-	-
rs132630312	X:69957093	C/T		non coding transcript exon variant	-	-	-	-	-	-	-
rs132630313	X:69957096	C/T		non coding transcript exon variant	-	-	-	-	-	-	-
rs132630314	X:69957097	G/**A**/T		non coding transcript exon variant	-	-	-	-	-	-	-
rs132630314	X:69957097	G/A/**T**		non coding transcript exon variant	-	-	-	-	-	-	-
rs727504649	X:69957104	A/C		non coding transcript exon variant	-	-	-	-	-	-	-
rs876657640	X:69957107	A/T		non coding transcript exon variant	-	-	-	-	-	-	-
rs1057521131	X:69957124	G/C		non coding transcript exon variant	-	-	-	-	-	-	-
rs727505013	X:69957133	G/A		non coding transcript exon variant	-	-	-	-	-	-	-
rs397516662	X:69957087	C/T		missense variant	R/C	**0**	**0.183**	**22**	**0.566**	**0.59**	**0.144**
rs132630312	X:69957093	C/T			R/C	**0**	**0**	**22**	**0.452**	**0.548**	**0.144**

				missense variant							
rs132630313	X:69957096	C/T		missense variant	R/C	0	0.02	23	0.551	0.513	0.224
rs132630314	X:69957097	G/**A**/T		missense variant	R/H	0.01	0.865	25	0.54	0.754	0.224
rs132630314	X:69957097	G/A/**T**		missense variant	R/L	0.04	0.394	23	0.518	0.668	0.224
rs727504649	X:69957104	A/C		missense variant	K/N	0.01	0.95	24	0.381	0.708	0.18
rs876657640	X:69957107	A/T		missense variant	R/S	0.01	0.879	25	0.381	0.705	0.088
rs1057521131	X:69957124	G/C		missense variant	G/A	0.05	0.995	23	0.493	0.751	0.557
rs727505013	X:69957133	G/A		splice donor variant	-	-	-	-	-	-	-
rs397516664	X:70023246	G/**C**/T		splice region variant intron variant	-	-	-	-	-	-	-
rs397516664	X:70023246	G/C/**T**		splice region variant intron variant	-	-	-	-	-	-	-
rs397516665	X:70027876-70027911	TGGACCCAATGGCCCTCC... /-		inframe deletion	PGPNGPPGPPGPP/P	-	-	-	-	-	-

rs397516666	X:70027883-70027918	AATGGCCCTCCAGGACCC.../-		inframe deletion	NGPPGPPGPPGP/-	-	-	-	-	-	-
rs397516667	X:70027892-70027919	CCAGGACCCCCAGGACCT.../-		frameshift variant	PGPPGPPGPQ/X	-	-	-	-	-	-
rs397516668	X:70027902-70027919	CAGGACCTCCAGGACCCC/-		inframe deletion	PGPPGPQ/Q	-	-	-	-	-	-
rs397516671	X:70027937	C/T		missense variant	P/S	0.02	0.999	26	0.527	0.873	0.492
rs1064793104	X:70027942-70027959	GATTCCTGGAATTCCAGG/-		inframe deletion	GIPGIPG/G	-	-	-	-	-	-
rs1057520742	X:70027947	C/T		missense variant	P/L	0.06	0.999	24	0.622	0.905	0.605
rs132630315	X:70027956	C/T	?	missense variant	P/L	0.05	0.999	24	0.735	0.883	0.704
rs876657685	X:70027978-70028013	ACCTGGTCCTCCAGGTCC.../-		inframe deletion	PPGPPGPPGPQGP/P	-	-	-	-	-	-
rs876657686	X:70027989-70028006	CAGGTCCTCCTGGTCCTC/-		inframe deletion	PGPPGPQ/Q	-	-	-	-	-	-
rs397516670	X:70027993-70028027	TCCTCCTGGTCCTCAAGG.../-		frameshift variant	GPPGPQGPPGLQG/GX	-	-	-	-	-	-
rs132630316	X:70028001	G/C		missense variant	G/A	0.02	0.999	27	0.978	0.989	0.893
rs727503007	X:70028006	C/T		stop gained	Q/*	-	-	-	-	-	-

rs886039344	X:70028037	G/A		splice donor variant	-	-	-	-	-	-	-
rs886039466	X:70029503	G/A		splice acceptor variant	-	-	-	-	-	-	-
rs397516672	X:70029527	C/T		stop gained	R/*	-	-	-	-	-	-
rs886042183	X:70029538	G/T		missense variant<br>splice region variant	Q/H	0.02	0.967	29	0.469	0.75	0.331
rs879255552	X:70030482	A/T		missense variant	H/L	0	0.998	28	0.926	0.977	0.474
rs1064793105	X:70030491	G/A		missense variant	G/D	0	0.999	28	0.927	0.978	0.331
rs727504417	X:70030493	C/**G**/T		missense variant	Q/E	0.01	0.784	24	0.66	0.966	0.43
rs727504417	X:70030493	C/G/**T**		stop gained	Q/*	-	-	-	-	-	-
rs1057517882	X:70030496	G/C		missense variant	G/R	0.02	0.923	32	0.808	0.968	0.627
rs879255611	X:70030503	C/A		missense variant	A/E	0.01	0.754	25	0.815	0.955	0.43
rs727503008	X:70033413	T/-		frameshift variant	V/X	-	-	-	-	-	-

rs397516675	X:70033426	G/**A**/T		stop gained	W/*	-	-	-	-	-	-
rs397516675	X:70033426	G/A/**T**		missense variant	W/C	0	1	35	0.951	0.988	0.404
rs397516676	X:70033426	G/-		frameshift variant	W/X	-	-	-	-	-	-
rs387907197	X:70033430	C/T		missense variant	R/C	0	0.968	28	0.817	0.953	0.065
rs1057517731	X:70033431	G/T		missense variant	R/L	0.02	0.329	26	0.697	0.901	0.065
rs879255551	X:70033469	C/T		missense variant	R/C	0	0.997	29	0.917	0.882	0.575
rs876657641	X:70033470	G/**A**/C		missense variant	R/H	0	0.992	34	0.821	0.881	0.389
rs876657641	X:70033470	G/A/**C**		missense variant	R/P	0	0.998	34	0.898	0.882	0.575
rs397516677	X:70033475	G/A		missense variant	G/R	0	1	33	0.948	0.985	0.675
rs886042021	X:70033476	G/A		missense variant	G/E	0	1	32	0.949	0.983	0.542
rs397516679	X:70033499	G/A		missense variant	G/S	0.01	1	28	0.958	0.996	0.81
rs397516681	X:70033506	A/G		missense variant	Y/C	0	0.999	29	0.969	0.977	0.799

rs876657642	X:70033515	A/C		missense variant	Y/S	0	0.998	29	0.968	0.945	0.81
rs727503009	X:70033521	A/**G**/T	?	missense variant	Q/R	0.01	0.986	27	0.918	0.987	0.808
rs727503009	X:70033521	A/G/**T**	?	missense variant	Q/L	0	0.978	28	0.955	0.987	0.808
rs1131691566	X:70033526	G/T		stop gained<br><br>splice region variant	E/*	-	-	-	-	-	-
rs1064793106	X:70033527	A/G		missense variant<br><br>splice region variant	E/G	0	1	35	0.758	0.842	0.297
rs1085307599	X:70035364	T/C		missense variant	Y/H	0	0.966	27	0.927	0.978	0.566
rs1057518211	X:70035365	A/C		missense variant	Y/S	0	0.786	30	0.929	0.981	0.694
rs727503010	X:70035381	C/-		frameshift variant	D/X	-	-	-	-	-	-
rs876657687	X:70035393	T/**C**/G		synonymous variant	Y	-	-	-	-	-	-
rs876657687	X:70035393	T/C/**G**		stop gained	Y/*	-	-	-	-	-	-
rs397516682	X:70035394	G/T		stop gained	E/*	-	-	-	-	-	-

rs727503011	X:70035424	C/T		stop gained	Q/*	-	-	-	-	-	-
rs142948132	X:70035434	G/**A**/C/T		missense variant	R/H	0.19	0.951	27	0.769	0.792	0.31
rs142948132	X:70035434	G/A/**C**/T		missense variant	R/P	0.2	0.974	27	0.843	0.863	0.399
rs142948132	X:70035434	G/A/C/**T**		missense variant	R/L	0.14	0.832	27	0.788	0.812	0.31
rs132630321	X:70035446	C/T		missense variant	T/M	0	0.954	24	0.875	0.873	0.519
rs132630317	X:70035478	G/**A**/T		missense variant	A/T	0	0.997	26	0.887	0.981	0.687
rs132630317	X:70035478	G/A/**T**		missense variant	A/S	0.17	0.996	24	0.726	0.979	0.482
rs876657639	X:70035500	C/T		missense variant	A/V	0.01	0.99	27	0.833	0.872	0.331
rs886039347	X:70035502	C/T		missense variant	R/W	0	0.915	27	0.749	0.948	0.17
rs132630320	X:70035505	C/G		missense variant	Q/E	0.03	0.969	24	0.783	0.959	0.18
rs397516654	X:70035527	T/C		missense variant	V/A	0.07	0.598	24	0.666	0.926	0.119
rs781394318	X:70035556	A/G		missense variant	K/E	0.03	0.041	25	0.591	0.824	0.028

rs780582849	X:70035570	C/**A**/T		missense variant	F/L	**0**	**0.967**	**26**	**0.938**	**0.934**	**0.784**
rs780582849	X:70035570	C/A/**T**		synonymous variant	F	-	-	-	-	-	-
rs749830948	X:70035577	G/**A**/T		missense variant	A/T	**0.03**	**0.652**	**26**	**0.771**	**0.831**	**0.298**
rs749830948	X:70035577	G/A/**T**		missense variant	A/S	**0.46**	**0.186**	**22**	**0.479**	**0.701**	**0.104**
